# Sample preparation of *Drosophila melanogaster* larvae for MALDI imaging

**DOI:** 10.1007/s00216-025-06289-x

**Published:** 2026-01-06

**Authors:** Marius Herbst, Marie A. König, Katharina Gutbrod, Johannes Volk, Peter Dörmann, Margret H. Bülow, Marianne Engeser

**Affiliations:** 1 Kekulé-Institute for Organic Chemistry and Biochemistry, University of Bonn, Bonn, Germany; 2 Life & Medical Sciences Institute (LIMES), University of Bonn, Bonn, Germany; 3 Institute of Molecular Physiology and Biotechnology of Plants (IMBIO), University of Bonn, Bonn, Germany; 4 Membrane Contact Sites, CURE3D Research Lab, Clinic for Cardiac Surgery, University Hospital Düsseldorf, Düsseldorf, Germany

**Keywords:** MALDI imaging, *Drosophila melanogaster*, Phospholipids, Triacylglycerides

## Abstract

Mass spectrometry-based lipidomics has emerged as a crucial field for unraveling the complexity of biological systems through comprehensive profiling of lipid species with unparalleled sensitivity and specificity. Among the available techniques, matrix-assisted laser desorption/ionization imaging mass spectrometry (MALDI imaging, or MALDI-IMS) stands out as a powerful spatial lipidomics tool, enabling the visualization of distribution and localization of individual lipid classes and species directly within biological tissues. The spatial context of lipids provided by MALDI imaging is especially valuable for studying nutritional and developmental processes in heterogenic tissues. The reliability and quality of MALDI imaging data critically depend on sample preparation, which must preserve both tissue morphology and analyte integrity. Here we introduce a sample preparation protocol for *Drosophila melanogaster* larval tissue, addressing key limitations of standard protocols such as thin-sectioning. Owing to the small size of *Drosophila* tissues and the flat morphology of organs such as the fat body, our approach enables direct imaging of the inner organs of the larvae without sectioning. This not only streamlines the workflow and minimizes tissue disruption but also allows simultaneous analysis of all major internal organs in a single measurement. Our approach not only facilitates high-quality MALDI-IMS data acquisition from complex larval tissues but also enables the localization of a broad spectrum of lipid species complementary to data gained by established techniques like liquid chromatography mass spectrometry (LC-MS). These advances position MALDI imaging, when coupled with our optimized preparation workflow, as an indispensable technique for spatially resolved lipidomics studies in small model organisms such as *Drosophila* larvae.

**Graphical Abstract:**

**Figure:**
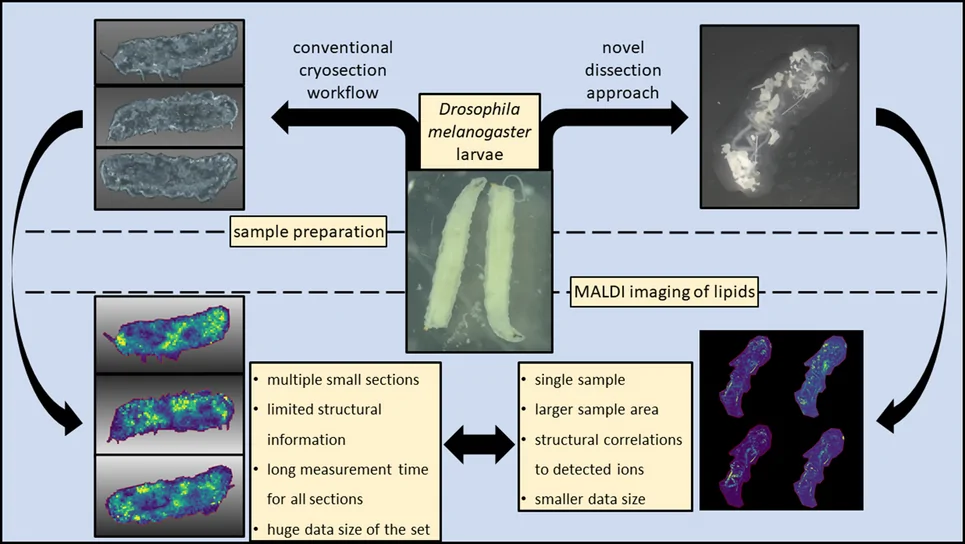

**Supplementary Information:**

The online version contains supplementary material available at https://doi.org/10.1007/s00216-025-06289-x.

## Introduction

 Matrix-assisted laser desorption/ionization (MALDI) imaging is a modern mass spectrometry application with surging interest in many research fields such as neurology, cancer, and drug research [1]. Besides mammalian tissues, it can be applied to plants [2] and insects [3] as MALDI in general covers a large mass range and is favourable for many kinds of analytes such as metabolites, lipids, peptides, and proteins [4]. The extension to MALDI imaging makes it possible to visualize the distribution of these compounds in different biological tissue samples. The images of specific compound localizations are referred to as ion intensity maps.

Without the need for molecular labels, the sample preparation, while highly critical, is in most cases straightforward and can be easily adapted to the analyte of interest. With modern instrumentation, a spatial resolution of 20 to 50 µm is the standard [5] but even smaller step sizes below 10 µm are possible [6]. However, this comes at the cost of measurement speed. For biological tissue samples, a cryotome is used to create thin sections with an optimal thickness of 10 µm [7]. This process requires some practice and skill as the frozen tissue slices are fragile and delicate to handle. For inhomogeneous biological samples that contain both rigid and soft parts, lengthy optimization is required. Additionally, the process of freezing and thawing of the tissue can cause harm and will result in damaged samples which in turn will produce poor signal intensities. Embedding the sample is a measure of protection against damage and makes small samples easier to handle. Yet, successful MALDI imaging measurements require considerable experience and care.

The fruitfly *Drosophila melanogaster* is established as a model organism in biological research for over a century. Since the work of Thomas Hunt Morgan in the early 1900 s, which laid the foundation for the chromosome theory of inheritance, *Drosophila* has played a pivotal role in numerous scientific breakthroughs spanning genetics, developmental biology, and neuroscience. Its sustained relevance arises from several advantages: a short life cycle, high fecundity, ease of genetic manipulation, and well-conserved metabolic pathways with higher organisms, including humans. These features, along with a fully sequenced and annotated genome, have facilitated large-scale, hypothesis-driven studies and made *Drosophila* especially adaptable to the demands of modern analytical techniques such as imaging mass spectrometry. *Drosophila*’s organs and metabolic pathways responsible for lipid synthesis, storage, and regulation share strong functional, anatomical, and genetic parallels with mammals. The modelling of metabolic diseases and nutrient-dependent changes in organ lipid distribution has become an essential component of modern lipidomics research. By employing model organisms such as *Drosophila melanogaster*, researchers can investigate the genetic and environmental factors contributing to metabolic disorders, mirroring many aspects observed in humans due to highly conserved metabolic pathways [8–10].

MALDI imaging has been employed to visualize surface lipids on whole flies [11], the effects of obesity induced by a high sugar diet on lipids in the adult fat body [9], lipids in the outer layer of egg chambers [12] and in cryosections of the brain [13] or whole adult flies [3, 14–16]. Here we sought to develop a method that would allow lipid analysis by MALDI imaging in whole dissected preparations of *Drosophila* larvae and compare them to larval cryosections.

In this study, we utilize MALDI imaging to visualize the distribution of different lipid classes in the organs, especially in the fat body of wild-type *Drosophila* larvae. We compare the ion intensity maps of classically embedded sample thin sections with a novel sample preparation method for MALDI imaging of *Drosophila* larvae. The main focus will be the detection and localization of endogenous lipids in wild-type larvae. This may serve as a starting point for a better understanding of metabolic pathways in genetically modified *Drosophila* larvae or in larvae challenged by a specific diet.

**Fig. 1:**
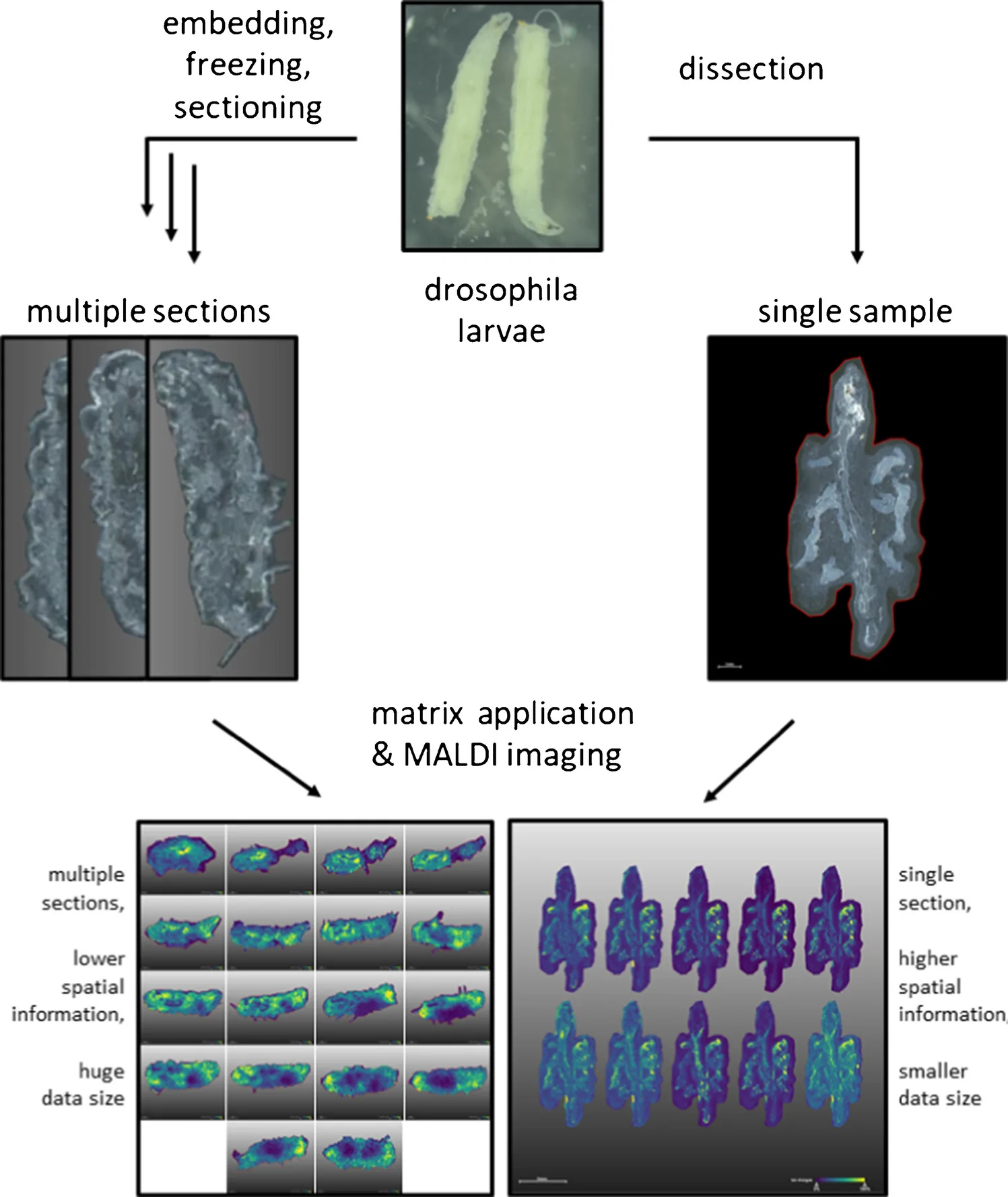
Schematic dual MALDI imaging workflow for both thin sections and dissected samples. While the samples have to be embedded, frozen, and then carefully sectioned in a cryotome, the dissection shortens the preparation time significantly. Afterwards, the steps are identical, but overall dissections decrease measurement time and data size

Lipids have many structural, metabolic, and regulatory tasks in an organism. Structurally, lipids are fundamental components of cellular membranes, determining flexibility and maintaining membrane fluidity and integrity. Key membrane lipids such as phospholipids, sphingolipids, and sterol lipids determine membrane architecture and modulate the activity of proteins within the membrane by affecting the local biophysical environment. Storage lipids like triacylglycerides are employed to store energy and carbon derived from metabolism. Regulatorily, certain lipids directly influence membrane protein function, cell signalling, gene expression, and membrane dynamics. Regulatory lipids, including diacylglycerol, phosphatidic acid, phosphoinositides, and sterols, can act as anchoring sites for partner proteins, modulate protein conformations, serve as signalling molecules, and create microdomains that impact processes like membrane fusion [17]. A dysregulated metabolism can lead to many diseases like the metabolic syndrome, including obesity and type 2 diabetes [3, 18]. *Drosophila* larvae play an important role in the study of lipids in animals, as they have a fast reproductive cycle and are easy to cultivate in large numbers. They serve as a model system for many research questions regarding metabolism and diseases [3]. For these small organisms, the challenges of sample preparation will be discussed, and a robust straightforward protocol will be established. Different embedding media were tested to optimize the procedure of sectioning. Additionally, a novel sample preparation method is presented that enhances the spatial information in comparison to the thin sections and reveals precious information about lipid distribution in *Drosophila* larvae (Fig. 1). Lipid assignment for data analysis was further refined with LC-MS measurements of dissected larvae and of isolated fat body.

## Materials and methods

### Sample collection

The *Drosophila melanogaster* flies (Genotyp w1118, Bloomington stock collection # 6326) were reared on a standard cornmeal–sucrose–yeast food (130 g yarn agar, 248 g baker’s yeast, 1223 g cornmeal, and 1.5 L sugar beet syrup in 20 L distilled water) in a 25 °C incubator with a 12-h light–dark cycle until they were utilized for experimental purposes. For cryosectioning experiments, eggs were collected by setting up cages with an equal number of males and females maintained at 25 °C with a 12-h light–dark cycle. Eggs were collected over a period of 24 h using apple juice agar plates (2% agar, 2.5% sucrose, 25% apple juice in double distilled water, and 10% nipagin in ethanol in a 1:1 ratio with a portion of yeast paste) (baker’s yeast mixed with water) on top. After 24 h of egg collection, the plates were incubated at 25 °C with a 12-h light–dark cycle for 72 h. For dissection and LC-MS experiments, flies were reared on a standard cornmeal–sucrose–yeast food (130 g yarn agar, 248 g baker’s yeast, 1223 g cornmeal, and 1.5 L sugar beet syrup in 20 L distilled water) in a 25 °C incubator with a 12-h light–dark cycle until they were utilized for experimental purposes. Early third instar larvae were collected based on the morphology of the mouth hook, anal papilla, anterior and posterior spiracles, and the orange colouring of the latter.

### Cryosections of whole larvae

Besides OCT medium, aqueous solutions of 2, 4, 8, and 10% carboxymethylcellulose (CMC) were tested for the embedding. The living larvae were placed inside a small drop of embedding solution within a plastic mold and manually aligned. This small droplet surrounding the larvae was frozen within seconds by placing the mold on dry ice. Then, the mold was filled completely with embedding solution, preventing air bubble formation at all times. After shock freezing with dry ice, frozen samples were stored at −80 °C until cryosectioning. The sample block was mounted onto the specimen disc. A Leica CM 3050 S cryostat was used to create thin sections. In our hands, 20 µm thickness yielded best results with respect to tissue integrity and signal intensity. The sections were placed onto the conductive side of indium tin oxide (ITO)–coated glass slides. Thoroughly thawing them from the back flattens the samples and prevents damage of the tissue when it is frozen again for storage at −80 °C until further procedures [19]. The slides were placed in a desiccator and dried under vacuum for 2 h either directly after sectioning or after they were taken out of the freezer to prepare them for matrix application and measurement.

### Sample dissection

By carefully dissecting the inner organs of *Drosophila* larvae, we separated them from the thick cuticle. To this end, larvae were washed in PBS to remove residual food from their surface and dissected in PBS under a Zeiss Stemi305 microscope. The larval cuticle was opened at the posterior end without rupturing the gut. Larvae were fixed at the cuticle above the mouth hooks, and the inner organs were released by inside-out preparation of the cuticle. Afterwards, the tissues were transferred to the ITO slide in a droplet of PBS, quickly arranged on the slide, and fully dried under vacuum. For method optimization, additional steps were added: (A) fixing the samples in 4% PFA in PBS at room temperature for 1 h, followed by three washing steps with PBS for 15 min after dissection. (B) Adding a washing step of the tissue before placing it on the slide with either ddH_2_O or EtOH. For that, a simple short dipping of a maximum of 3 s in either ddH_2_O or EtOH was done. (C) Dissecting the larvae directly on the slide. All dissected samples were carefully rearranged on the slide to prevent overlap of tissue. This is only possible for a limited time when the tissue is still enclosed in liquid and will otherwise result in tissue crumbling and rupture.

### MALDI matrix application

HCCA was chosen as a suitable matrix for lipids. For both sample types, HCCA was sprayed on the slides with a TM sprayer from HTX imaging (HTX Technologies, LLC) at a concentration of 7 mg/mL in a mixture of acetonitrile, H_2_O, and trifluoroacetic acid (volume ratios, 50:50:0.2). The flowrate was 0.1 mL/min, and ten even layers of matrix were applied for both sample types. Later, the number of matrix layers was reduced to four for the dissected samples to improve the signal quality of the lipids and reduce the intensity of the matrix signals.

### MALDI instrumentation and data analysis

A Bruker ultrafleXtreme MALDI-TOF/TOF mass spectrometer equipped with a smartbeam™-II laser was used for the MALDI imaging measurements. The setup of the imaging run was done with the flexImaging software in combination with the flexControl software, both from Bruker. The acquired spectra covered a mass range of *m/z* 300 to 1300 measured in positive reflection mode. Spectra from 500 laser shots at a repetition rate of 1000 Hz were accumulated per spot. The spatial resolution was typically set to 30 µm × 30 µm. The instrument was calibrated before measurement by applying a spot of red phosphorus next to the sample region and using the detected P-clusters for mass calibration.

 The acquired MALDI imaging data was analyzed with SCiLs Labsoftware (Bruker). All data sets were normalized to the total ion current (TIC), and the ion-intensity maps are depicted. For segmentation, the designated pipeline of SCiLS Lab software was used. Herein, hundred relevant peaks are determined by the software via orthogonal matching pursuit (OMP) using every 16th spectrum. Then, the peaks are moved to the local maxima, and afterwards segmentation is done with bisecting k-means and medium denoising.

Signal assignments are matched with the LIPID MAPS® Structure Database (LMSD) [20]. The ion–intensity maps depicted herein belong to lipid signals previously reported for adult *Drosophila* by Merkler et al. [3].

### Lipid extraction and LC–MS measurements of homogenized larval tissue

For complementary data, two batches of 30 larvae were prepared for LC-MS studies. One half of the larvae were simply dissected, while from the other half, only the fat body was collected after dissection. Afterwards, the tissues were homogenized with glass beads in 400 µL H_2_O using the Precellys Evolution Touch Homogenizer with 3 cycles of 10 s homogenization at 5500 RPM and 20 s cool down. The volumes were separated into two aliquots and extracted according to Matyash et al. [21] with methyl-*tert*-butylether (MTBE). Lipid extracts were diluted 1:1000 with injection solvent composed of 7:2:1 (v/v/v) tetrahydrofuran (THF)/methanol/5 mM ammonium formate + 0.1% formic acid.

Chromatographic separation of lipids was carried out on a Supelcosil ABZ plus (100 × 2.1 mm, 3 µm particle size, Supelco) HPLC column with precolumn and using an Agilent 1260 Series HPLC system equipped with a flexible pump. The flow rate was maintained at 0.2 mL/min. The LC solvents were solvent A (3:2:5 (v/v/v) tetrahydrofuran (THF)/methanol/5 mM ammonium formate + 0.1% formic acid) and solvent B (9:1 (v/v) THF/5 mM ammonium formate + 0.1% formic acid). A gradient elution from 60% B to 100% B over 8 min was applied.

Lipid chromatography–mass spectrometric analysis was performed using an Agilent 6546 Q-TOF LC-MS system operating in positive ion mode. Data acquisition involved Auto-MS/MS with subsequent targeted scanning using Agilent MassHunter Qualitative Analysis software. Lipid molecular species were identified based on the characteristic fragment ions using the Agilent Lipid Annotator software.

## Results and discussion

In this study, we present two sample preparation methods for MALDI imaging of third instar wild-type *Drosophila* larvae. In the first approach, the larvae were cryosectioned by adapting an established protocol designed for imaging soft tissue samples. In the second method, the larvae were dissected under a microscope and arranged directly onto ITO slides. In both workflows, we aimed at recording the spatial distribution of endogenous lipids in wild-type *Drosophila* larvae.

### Sectioned *Drosophila* larvae

The body of the *Drosophila* larva has a tough, waterproof, chitin cuticle on the outside protecting the soft inner organs, predominantly fat bodies. This heterogeneity makes it complicated to create thin sections with intact morphology. To preserve sample integrity during sectioning, an embedding medium was used to create a hard embedding surrounding the larvae that was comparable to the cuticle in toughness. For embedding, OCT medium was tried as well as CMC at different concentrations of 2, 4, 8, and 10% (w/v). While attempting to preserve morphological features of the tough cuticle as well as the soft lipid-rich fat body, occasional rupturing of the cuticle occurred. From the two embedding media, CMC proved to be the better choice. Embedding in CMC at a relatively high concentration of 10% results in a hard mold which led to the lowest observations of cuticle rupture. With OCT, more fissions from sectioning became visible. The larval body measures only a few millimeters in length, resulting in correspondingly small thin sections. Although lateral sections might have provided more optimal anatomical views, longitudinal sectioning was chosen, as lateral cuts would have yielded an even greater number of smaller, more fragile sections.

In this fashion, a series of around 18 consecutive thin sections with 20 µm thickness were produced of a single larva. Alternating sections were stained with phalloidin-TRITC and BODIPY 493/503 to visualize bodywall muscle and fat body, respectively (see Figure S1 in the Supporting Information). The sections were subjected to MALDI imaging at a spatial resolution of 50 µm × 50 µm which took around 1 h of measurement time for each section.

**Fig. 2:**
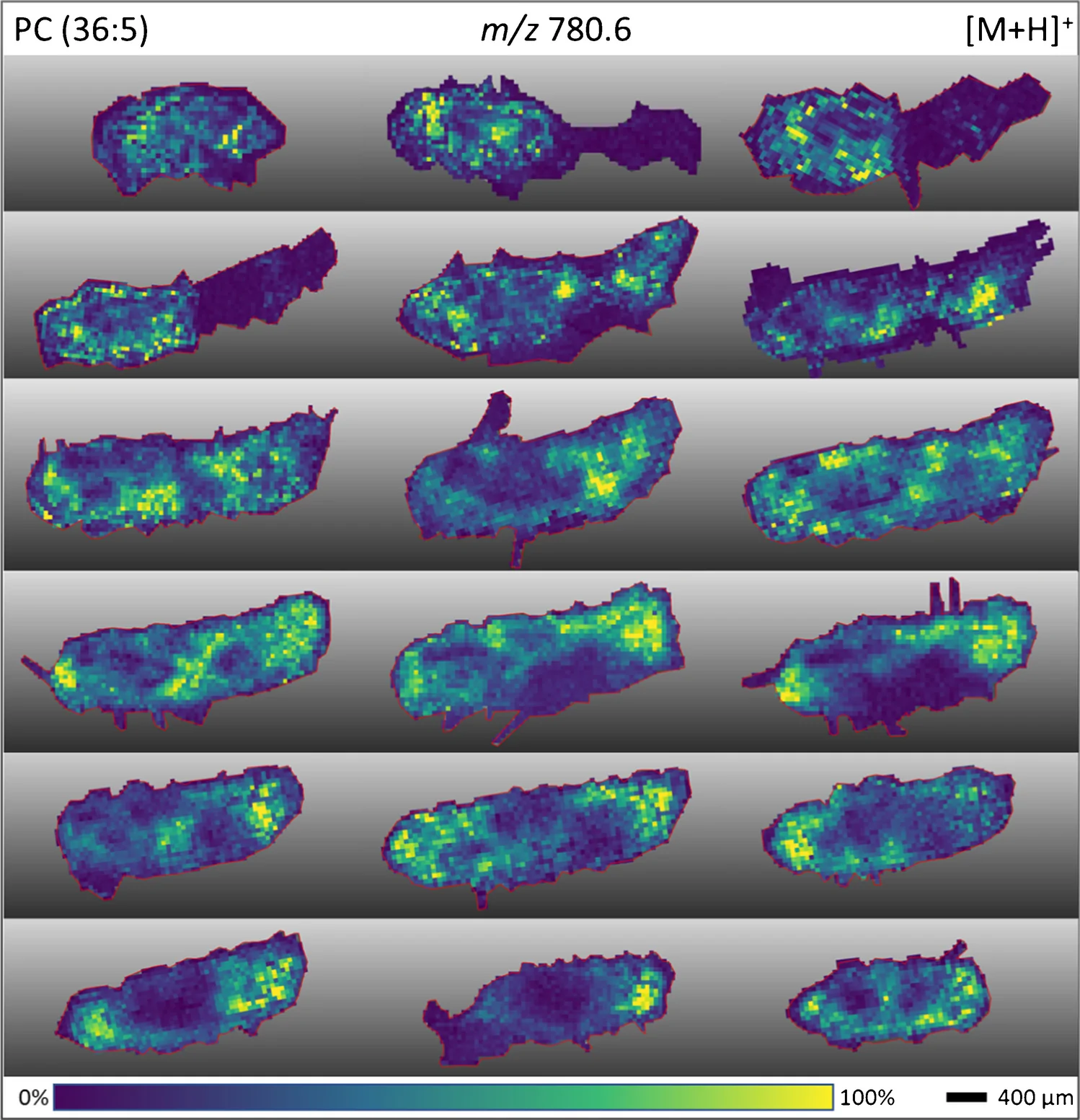
From left to right, top to bottom: ion intensity maps of PC (36:5) with m/z 780.6 [M + H]^+^ of consecutive longitudinal thin sections of a single larva measured with a spatial resolution of 50 µm × 50 µm

In the resulting mass spectra, dominant signals could be assigned to lipids, expectedly mostly phosphatidylcholines [22]. The ion intensity maps (Fig. 2) reveal distinct lipid signal distributions, yet the results remain inconclusive. The small sample size only allows for a limited number of pixels at step sizes usually achievable with our instrument, preventing the generation of highly detailed lipid distribution images and making different anatomical regions difficult to distinguish. Improving spatial resolution would require decreasing the pixel size; however, smaller pixel sizes reduce the number of ions detected per measurement point, lowering signal intensity. This trade-off also increases both measurement time and data volume. Further, a very small pixel size below 10 µm is only possible with modern high-end instruments. Taken together, these factors led us to develop a novel sample preparation method aimed at increasing the effective sample area while reducing measurement time.

### Dissected *Drosophila* larvae

Dissecting *Drosophila* larvae is an established method for staining experiments yielding optimal results in microscopy-based studies as the tough protective cuticle inhibits penetration with antibodies for immunostaining [23]. Here we used this approach for a novel MALDI imaging sample preparation protocol and compared it to MALDI imaging spectra from cryosections. The rationale was to reduce the sample preparation time, the number of collected spectra, and to be able to analyze the tissue of a whole larva in one measurement run.

**Fig. 3:**
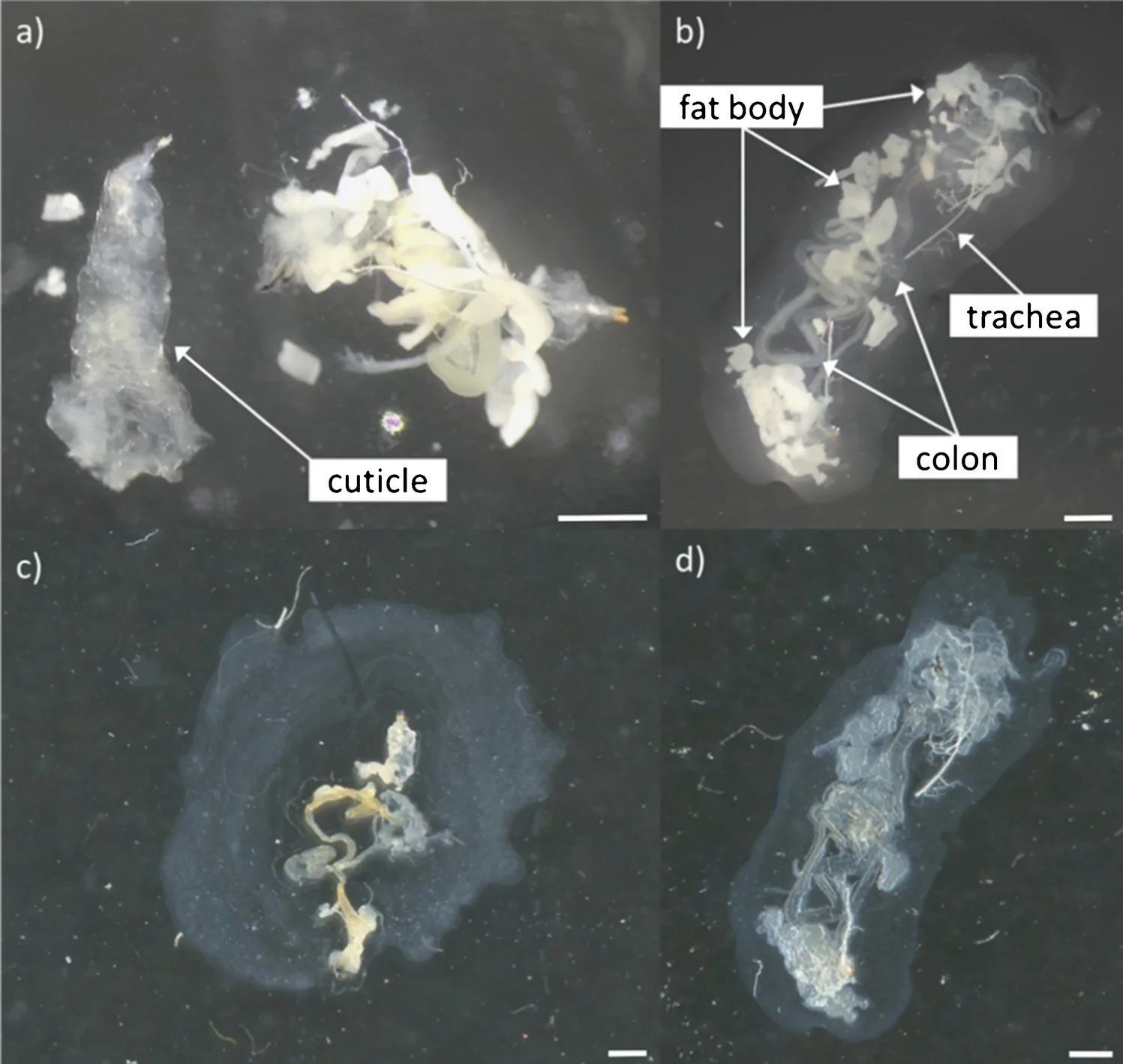
Optical images of dissected larvae. **a** Larva after dissection with organs separated from the cuticle. **b** Carefully rearranged tissue to avoid overlapping. Fat body can be recognized as the bright white mass, the gut as the bigger grey tube-like structure, and the trachea as the smaller, branched tube-like structure in the picture. **c** Dried ethanol-washed tissue with no visible fat body. **d** Dried water-washed tissue of **b** with still preserved fat body. All scale bars in this figure correspond to 200 µm

The dissection of the larvae was done in PBS under the microscope (Fig. 3a). An experienced researcher can dissect several larvae in a few minutes. Therefore, the dissection approach saves time that otherwise is spent on embedding, freezing, and sectioning a sample. Additionally, it can be quickly redone in case sufficient larvae are available. This method also comes with the beneficial side effect that it spreads out the inner organs of the larval body. This allows for a greater sample area that gives better spatial information. The different regions of fat body and gut can be better distinguished, and while the sample is still fresh on the slide, it can be rearranged to avoid overlapping regions of tissue (Fig. 3b).

In a first trial, we placed the dissected larvae on ITO-coated slides and stored them at −80 °C until analysis, which, however, resulted in low signal intensities. We then tested if PFA fixing would yield better results, but only observed some changes in relative signal intensities without enhancing overall spectra quality. However, the signal intensities improved when the samples were no longer frozen after dissection but were placed under vacuum immediately instead and measured without further delay. When the larva is just dissected and mounted on the ITO slide, the spectra show many dominant signals outside of the sample region, probably stemming from the PBS solution and resulting in strong ion suppression on the sample itself. Therefore, we considered that adding a washing step might remove buffer salts and enhance the spectral quality [24, 25]. EtOH was very aggressive towards the tissue, especially towards the fat body, and it was observed that most of the latter was dissolved after the washing step (Fig. 3c). Washing with EtOH thus dehydrates the tissue and leads to fast drying and brittle tissue and additionally destroys the fat body and washes out lipids [25]. However, using ddH_2_O for washing seemed to have the desired effect (Fig. 3d and S17 in the Supporting Information). Here, washing times should be kept at a minimum, as prolonged exposure, especially of the fat body, to the distilled water results in osmotic rupture of its cells. The measurements initially were done with ten layers of matrix, which resulted in very dominant matrix signals. To reduce these and enhance endogenous signals from the tissue, the number of matrix layers was reduced to four.

In summary, we found that direct processing of the dissected samples without freezing is critical for optimal signal detection. EtOH fixation damages fat body tissue and was subsequently avoided. The optimal procedure is thus the fresh dissection of samples on ITO-coated slides, washing with ddH_2_O, direct desiccation, and matrix application in four layers.

**Fig. 4:**
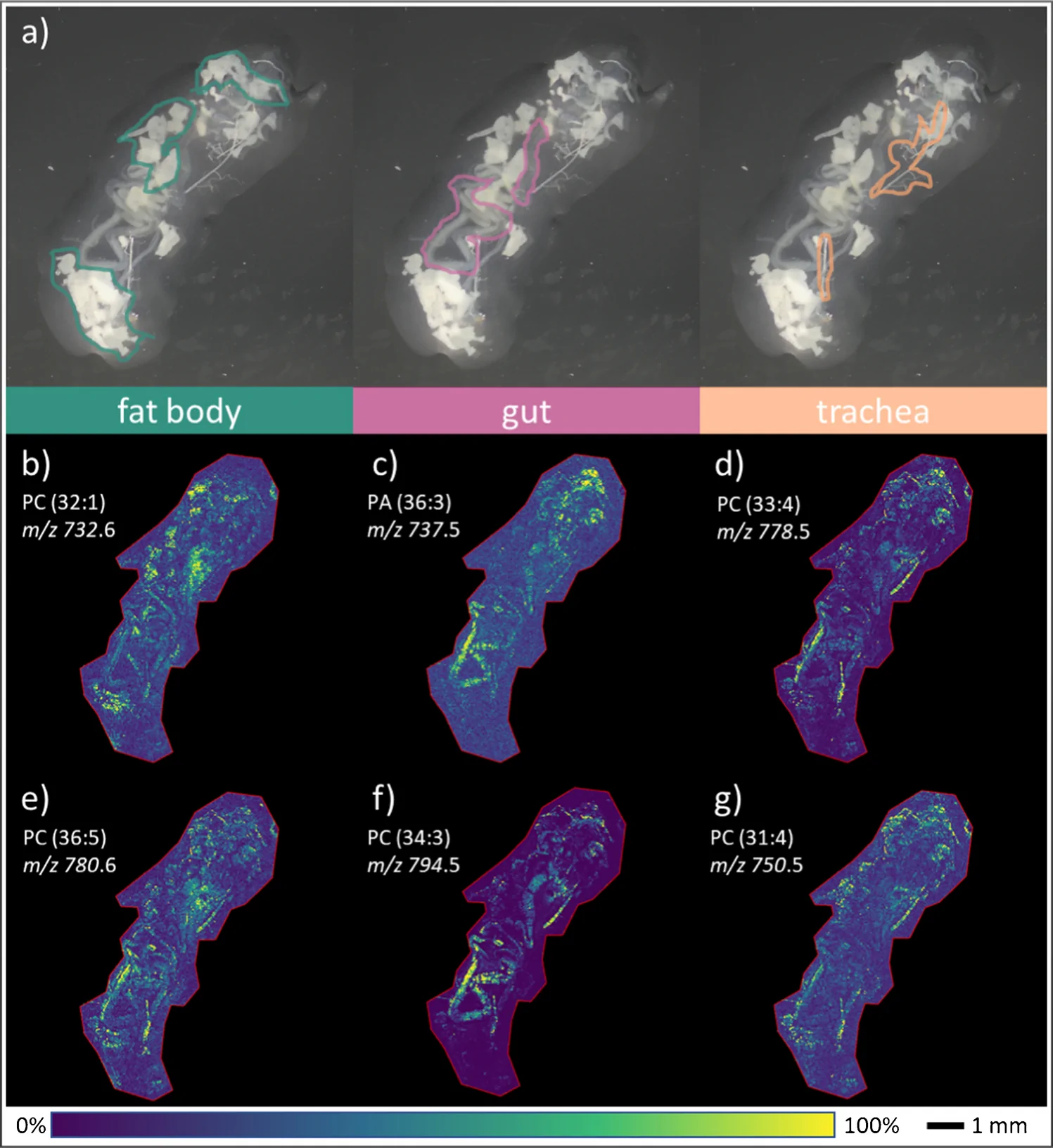
**a** Optical image of dissected sample as seen in Fig. 3d with marked regions of fat body (green), gut (pink), and trachea (orange). **b**–**g** MALDI imaging measured with a spatial resolution of 30 µm × 30 µm. Ion intensity maps of different lipids of a dissected larva highlighting different sample regions. **b** m/z 732.6 (PC 32:1), **c** m/z 737.5 (PA 36:3) or (PG 32:4), **d** m/z 778.5 (PC 33:4), **e** m/z 780.6 (PC 36:5), **f** m/z 794.5 (PC 34:3), and **g** m/z 750.5 (PC 31:4)

The new method greatly improved the quality of the spectra, and due to the larger sample areas, the ion intensity maps showed distinguishable distributions of different lipids in good resolution without the need for very small imaging pixel sizes, which can only be achieved by high-end mass spectrometers. Further, the new dissection sample preparation also reduced the number of measurements required for the observation of a complete larva to a single measurement. Albeit cuticle and hemolymph are left out, highly interesting inner organs like fat body and gut are nicely covered and depicted well (Fig. 4). The measurement time of a single run is 5 to 8 h for a dissected sample. It thus provides the complete and detailed coverage of the inner organs of a larva in less total measurement time, with less data size, and overall in a more straightforward procedure.

**Fig. 5:**
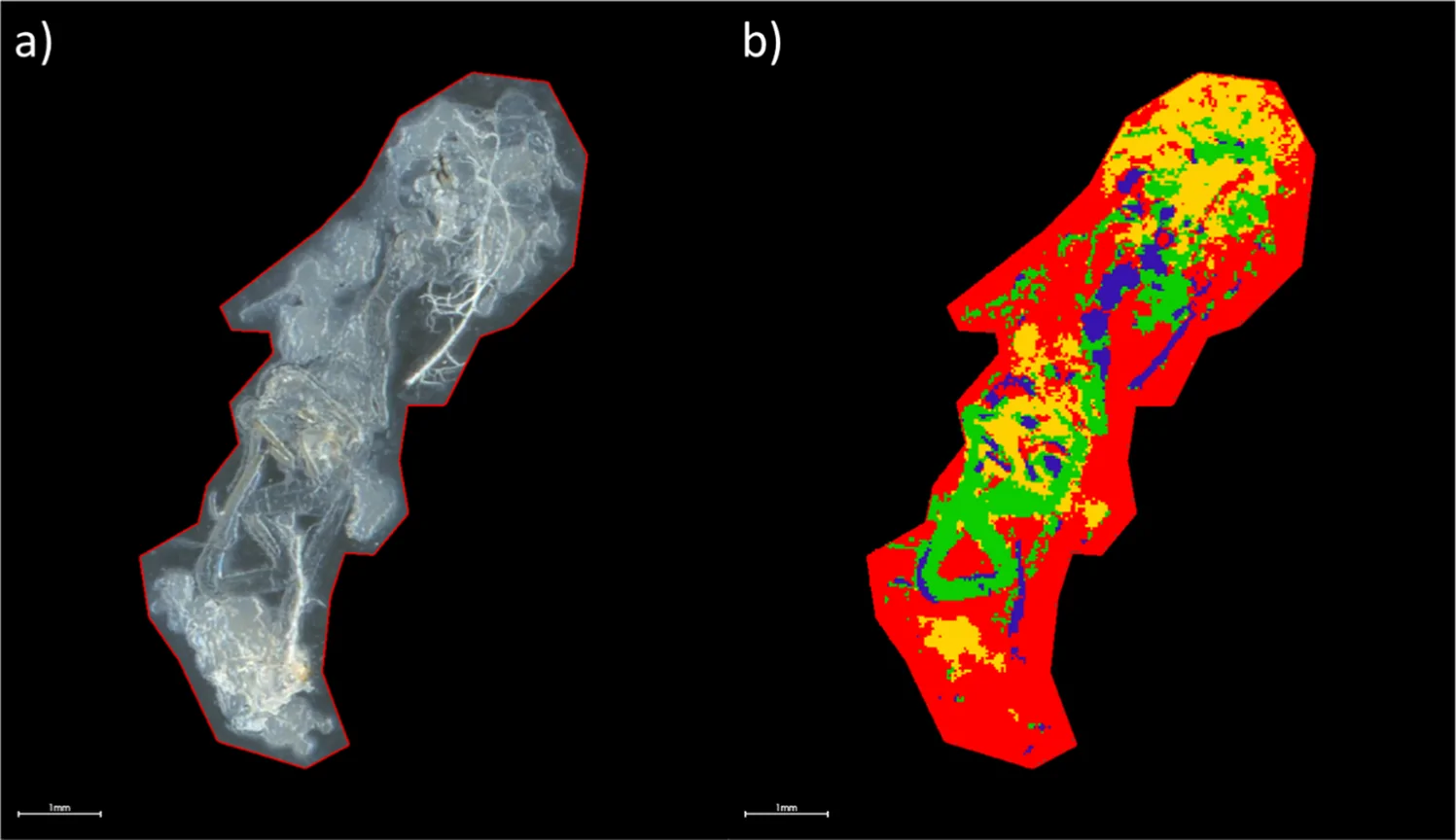
**a** Optical image of dissected *Drosophila* sample as seen in Fig. 3d. **b** Segmentation clusters in different colours that match tissue anatomy: fat body (yellow), gut (green), trachea/other (blue), and off sample (red)

 Using the segmentation pipeline implemented in the SCiLS Lab software for data analysis initially resulted in two clusters. These were then each split once again producing the yellow and red and the blue and green clusters depicted in Fig. 5. These cluster segments by and large represent the different tissue parts of fatbody, gut, trachea, and off-sample region (Figs. 4 and 5). It has to be noted though that the trachea cluster also includes parts of other areas in the gut and fat body regions. Additional *Drosophila* larvae dissections have been measured and analyzed similarly (see two additional examples in the Supporting Information, Figures S3-S16 including ion intensity box plots of *m/z*-values with high co-localization of the different cluster regions). Despite arranged differently on the slides in each case, cluster localization to the respective tissue parts clearly remains and show the robustness of the proposed sample preparation and also some specificity of phospholipid distribution for the different regions.

### LC–MS measurements of lipids from *Drosophila* larvae

**Fig. 6:**
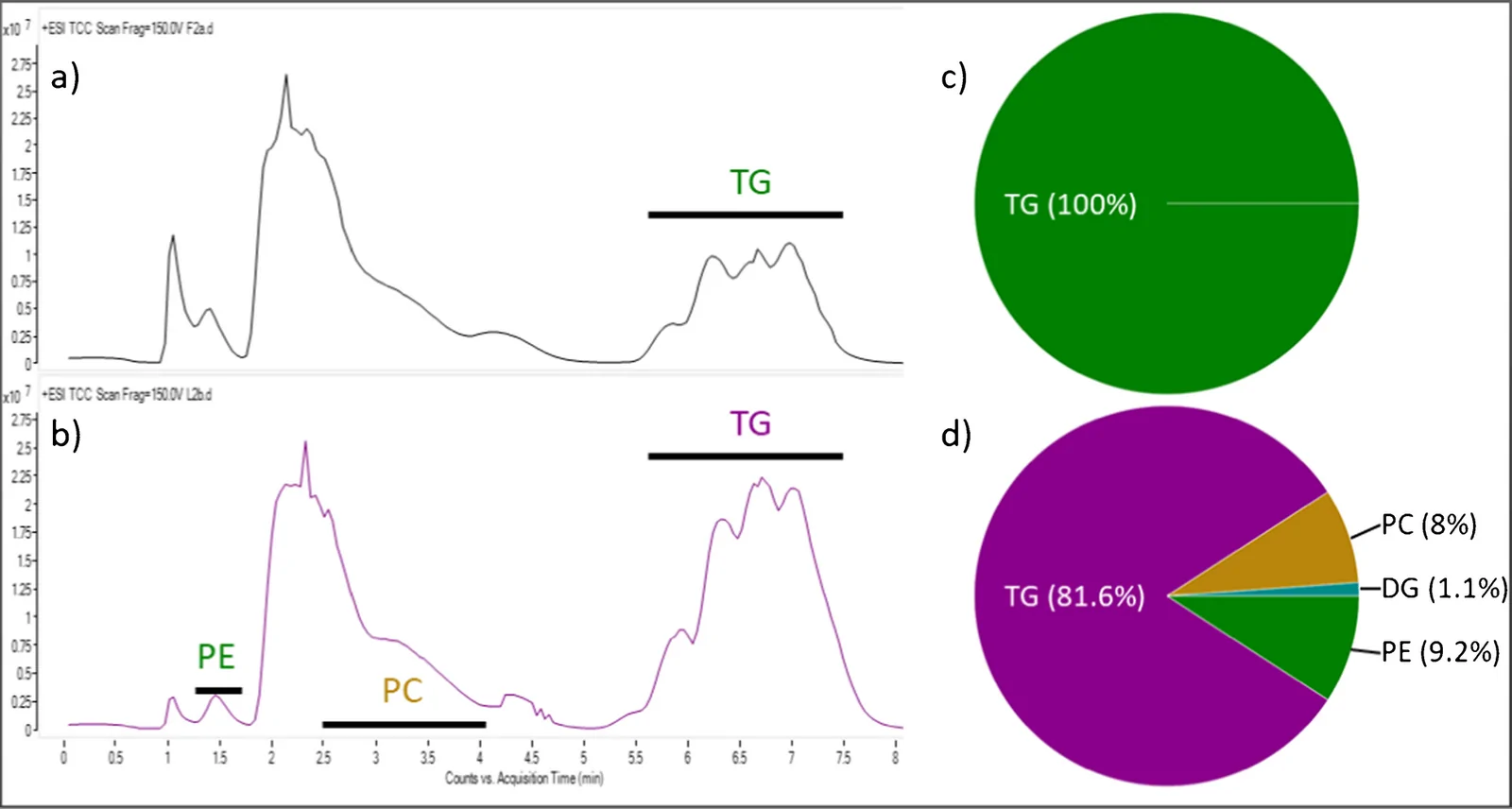
Summed signal intensities of molecular features detected in LC–MS measurements with marked retention time areas of identified lipid classes. **a** Total compound chromatograms (TCC) of fat body samples. **b** TCC of dissected larvae samples. Bar diagrams displaying the relative lipid composition of **c** fat body samples and **d** dissected larvae samples, based on identification of detected molecular features using Agilent Lipid Annotator

To further support lipid signal assignments in the imaging data, complementary LC-MS analysis was performed. Both dissected larvae as well as isolated fat body samples were subjected to lipid extraction followed by an untargeted lipidomics LC-MS/MS approach. The results reveal that triacylglyceride (TG) species are predominantly detected (Fig. 6), particularly fat body samples composed solely of adipose tissue. In the dissected larvae samples, also other lipids like phosphatidylcholines (PC) and phosphatidylethanolamines (PE) and one diacylglyceride (DG) species were found in good signal intensities. These results are in full accordance with our previously reported lipidomics data set of wild-type *Drosophila* larval fat body tissue in which TG was found to be the dominating lipid class, i.e. about 100 times more abundant than DG, phospholipids, and other lipid species [26].

### Lipid distributions

Signal *m/z*-values in the MALDI imaging data sets are assigned to lipids using the LIPID MAPS® Structure Database (LMSD) [20]. Afterwards, the proposed matches were compared with LC-MS/MS data and MALDI imaging signals reported for adult *Drosophila* [3, 14] (Tables 1 and 2). From this analysis, mainly phosphatidylcholines were identified in the MALDI imaging data. Note that the assignment to PCs is based on accurate mass data only. We therefore cannot differentiate between PC and PE lipids as we do not have tandem mass spectral data at this point. In our LC-MS/MS dataset, both PC and PE lipids have been identified on the basis of retention time and fragmentation data. Our ion intensity maps thus may show a superposition of both. For the sake of simplicity, we restricted the labels to PC herein. While odd-chain fatty acid lipids are known to be present in *Drosophila* [26, 27], the mass accuracy of our MALDI imaging data is not sufficient to unambiguously distinguish them from isobaric PC-O lipids [28].

**Table 1:** Measured masses of phospholipids in MALDI imaging of dissected *Drosophila* larvae with their respective matched lipid species according to LMSD [20]. Lipid signals detected and identified via LC-MS/MS experiments of this work or our previous results of extracted larvae fat body tissue are marked, as well as lipid signals found in previous MALDI imaging studies of adult *Drosophila* [3, 13, 14].

Phospholipids								
Measured mass	Theoretical mass	Delta	Name	Formula	Ion	Tissue region	LC–MS/MS	MALDI imaging
704.543	704.5225	0.0205	PC 30:1	C38H75NO8P	[M + H]^+^	Even, fat body	[26]	[3]
730.569	730.5381	0.0309	PC 32:2	C40H76NO8P	[M + H]^+^	Even, fat body	[26]	[3, 14]
732.584	732.5538	0.0302	PC 32:1	C40H78NO8P	[M + H]^+^	Even, fat body	This work, [26]	[3, 14]
737.474	737.4518	0.0222	PA 36:3	C39H71O8PK	[M + K]^+^	Gut		
742.509	742.4784	0.0306	PC 30:1	C38H74NO8PK	[M + K]^+^	Even	[26]*	
750.477	750.4471	0.0299	PC 31:4	C39H70NO8PK	[M + K]^+^	Tissue edges, trachea		
752.545	752.5225	0.0225	PC 34:5	C42H74NO8P	[M + H]^+^	Even, tissue edges	[26]	[3]
752.545	752.5201	0.0249	PC 32:2	C40H76NO8PNa	[M + Na]^+^	Even, tissue edges	[26]*	
754.559	754.5381	0.0209	PC 34:4	C42H76NO8P	[M + H]^+^	Even	[26]	[3]
756.563	756.5538	0.0092	PC 34:3	C42H79NO8P	[M + H]^+^	Even, gut	[26]	[3, 14]
758.595	758.5694	0.0256	PC 34:2	C42H80NO8P	[M + H]^+^	Even, gut	This work, [26]	[3, 14]
760.61	760.5851	0.0249	PC 34:1	C42H82NO8P	[M + H]^+^	Even, fat body	This work, [26]	[3, 13, 14]
768.524	768.494	0.03	PC 32:2	C40H76NO8PK	[M + K]^+^	Even	[26]*	
770.547	770.5097	0.0373	PC 32:1	C40H78NO8PK	[M + K]^+^	Even	[26]*	
772.553	772.5253	0.0277	PC 32:0	C40H80NO8PK	[M + K]^+^	Even	[26]*	
776.485	776.4627	0.0223	PC 33:5	C41H72NO8PK	[M + K]^+^	Tissue edges, trachea		
778.518	778.4784	0.0396	PC 33:4	C41H74NO8PK	[M + K]^+^	Tissue edges, trachea		
780.58	780.5538	0.0262	PC 36:5	C44H78NO8P	[M + H]^+^	Even, fat body	[26]	[3]
782.593	782.5694	0.0236	PC 36:4	C44H80NO8P	[M + H]^+^	Even	This work, [26]	[3, 13, 14]
784.615	784.5851	0.0299	PC 36:3	C44H83NO8P	[M + H]^+^	Even	[26]	[3, 13, 14]
794.514	794.5097	0.0043	PC 34:3	C42H78NO8PK	[M + K]^+^	Gut, fat body, trachea	[26]*	
796.558	796.5253	0.0327	PC 34:2	C42H80NO8PK	[M + K]^+^	Gut	[26]*	
798.573	798.541	0.032	PC 34:1	C42H82NO8PK	[M + K]^+^	Even	[26]*	
822.468	822.4471	0.0209	PE 40:10	C45H70NO8PK	[M + K]^+^	Gut		
824.587	824.5566	0.0304	PC 36:2	C44H84NO8PK	[M + K]^+^	Gut	[26]*	

^*^detected in ref. [26] as [M + H]^+^

**Table 2:** Measured masses of triacylglycerides in MALDI imaging of dissected Drosophila larvae with their respective matched lipid species according to LMSD [20]. Lipid signals detected and identified via LC–MS/MS experiments of this work or in [26] are marked, as well as lipid signals found in previous MALDI imaging studies of adult Drosophila [3, 14].

Triacylglycerides								
Measured Mass	Matched Mass	Delta	Name	Formula	Ion	Tissue region	LC–MS/MS	MALDI imaging
743.648	743.616	0.032	TG 42:1	C45H84O6	[M + Na]^+^	Even	This work, [26]*	[3, 14]
745.669	745.632	0.037	TG 42:0	C47H84O6	[M + Na]^+^	Gut	This work, [26]*	[14]
745.669	745.6341	0.0349	TG 44:3	C47H84O6	[M + H]^+^	Gut	This work, [26]*	
769.658	769.6317	0.0263	TG 44:2	C47H86O6	[M + Na]^+^	Trachea, even	This work, [26]*	[14]
771.676	771.6497	0.0263	TG 46:4	C49H86O6	[M + H]^+^	Gut	This work, [26]*	
771.676	771.6473	0.0287	TG 44:1	C47H88O6	[M + Na]^+^	Gut	This work, [26]*	[3, 14]
795.674	795.6473	0.0267	TG 46:3	C49H88O6	[M + Na]^+^	Gut, trachea	This work, [26]*	[14]
797.687	797.663	0.024	TG 46:2	C49H90O6	[M + Na]^+^	Gut, trachea	This work, [26]*	[3, 14]
799.703	799.681	0.022	TG 48:4	C51H90O6	[M + H]^+^	Even	This work, [26]*	
799.703	799.6786	0.0244	TG 46:1	C49H92O6	[M + Na]^+^	Even	This work, [26]*	[3, 14]
813.723	813.6943	0.0287	TG 47:1	C50H94O6	[M + Na]^+^	Even	This work, [26]*	
823.7	823.6786	0.0214	TG 48:3	C51H92O6	[M + Na]^+^	Gut	This work, [26]*	[3, 14]
825.71	825.6943	0.0157	TG 48:2	C51H94O6	[M + Na]^+^	Even	This work, [26]*	[3, 14]
827.734	827.7123	0.0217	TG 50:4	C53H94O6	[M + H]^+^	Even	This work, [26]*	
827.734	827.7099	0.0241	TG 48:1	C51H96O6	[M + Na]^+^	Even	This work, [26]*	[3, 14]
841.741	841.7256	0.0154	TG 49:1	C52H98O6	[M + Na]^+^	Even	This work, [26]*	
853.746	853.7256	0.0204	TG 50:2	C53H98O6	[M + Na]^+^	Even	This work, [26]*	[14]

^*^detected in ref. [26] as [M + NH_4_]^+^

 Four different major lipid distributions were apparent at first sight, i.e. predominantly in the fat body, the gut, around the tissue edges, or rather evenly distributed across the whole tissue (Fig. 4). Most of the abundant lipids belong to the latter, whereas some showed high signal intensities in specific regions. Similar regions of interest are obtained by segmentation analysis (Fig. 5; see also the box plots with co-localized signals in the Supporting Information). For example, PC (32:1) and PC (36:5) aggregated in the fat body although they could be found throughout the whole tissue. PC (34:3), PA (36:3), PC (34:2), PC (36:2), and PE (40:10) had high localization at the gut area. Signals belonging to PC (31:4), PC (34:5), and PC (32:2) were distributed more around the tissue edges. While that was also visible for PC (33:5), PC (33:4), and PC (31:4), they also had strong signal intensities at the trachea. This may give a hint at the role these lipids play in the organism. The fat body of *Drosophila* consists of cells derived by proliferation of mesoderm cells. The cells are rich in lipid droplets where the triacylglycerides are stored. The membranes of the fat body cells contain phospholipids like PC, as all eukaryotic cells [29]. In the larval fat body, PC play a role in lipid droplet size regulation. Under low levels of PC, lipid droplets fuse and TG accumulate, leading to inability of the tissue to mobilize storage lipids from lipid droplets [30].

**Fig. 7:**
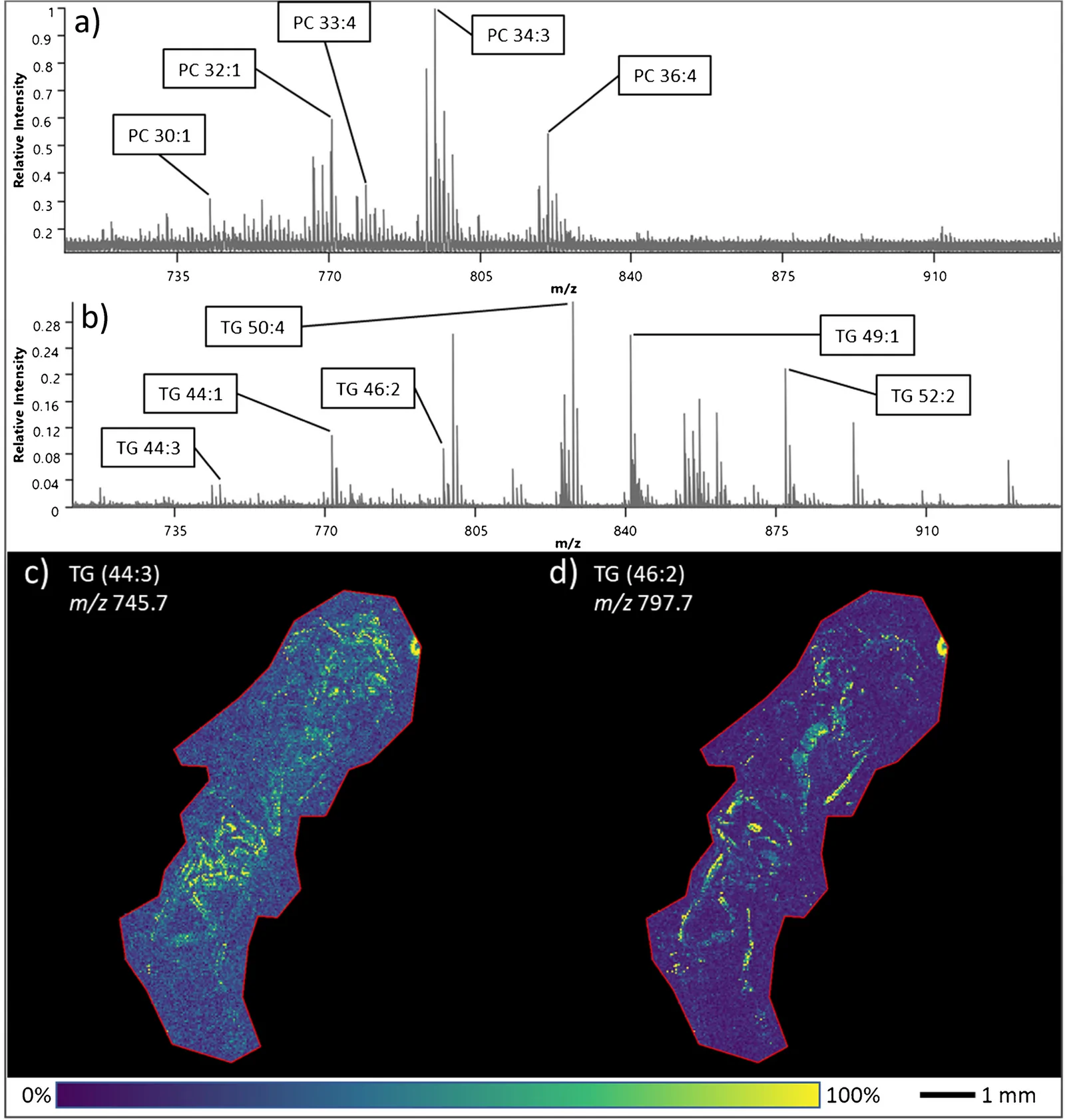
**a** Average MALDI mass spectrum showing dominant phosphatidylcholines. **b** Local MALDI mass spectrum showing suppressed TG signals. **c**, **d** Ion intensity maps of the two signals m/z 745.7 (TG (44:3)) with a relatively even intensity distribution over the tissue and m/z 797.7 (TG (46:2)) with strong intensities in the gut and trachea region. The respective optical image is shown in Figs. 4a and 5a

While triacylglycerides dominate the LC-MS/MS results, the MALDI imaging data mostly showed phosphatidylcholines due to severe ion suppression of neutral TGs in contrast to the more polar phosphatidylcholines [22, 31]. Still, some signals belonging to TGs are resolved in the averaged spectrum. When focusing on the local spectra of the regions where these signals had high intensity, it became clear that the TG species are present (Fig. 7) and not exclusively located in the fat body. The signals for TG (44:1), TG (44:3), TG (46:2), TG (46:4), and TG (48:3) were distributed strongly in the gut area, while TG (44:2) and TG (46:2) could be found at the trachea and the latter also in the gut. Other TG signals show a relatively even distribution with low intensities across the whole sample area. This further highlights the sensitivity of MALDI imaging in the detection of both polar and neutral tissue-specific lipids.

TG are abundant in fat body tissue and also make up a large portion of gut lipids. The gut, especially the midgut, contains numerous lipid droplets and serves as a specialized lipid reservoir [32]. By contrast, it is not known that TG are stored in the trachea, with the exception of specific regions such as the spiracles. Thus, our sensitive method is suitable to detect the spatial distribution of lipids in individual organs, driving the discovery of lipid stores that have not been described yet.

## Conclusion

In this study, we developed a sectioning protocol for *Drosophila* larvae for MALDI imaging purposes. Additionally, we present a novel preparation method for *Drosophila* larvae for MALDI imaging that bypasses the long preparation steps of cryosectioning and reduces measurement time and data size while enabling the measurement of a whole larva in a single run. The spatial information is greatly improved by expanding the inner organs of a specimen and therefore showing valuable information at a standard spatial resolution.

With this new approach, MALDI imaging allowed for the detection of a broad spectrum of lipids in wild-type *Drosophila* larvae and their distribution patterns in the organism. Expectedly, the majority of intense signals could be assigned to phosphatidylcholines or phosphatidylethanolamines and a single phosphatidic acid species (PA 36:3). Triacylglycerides (TG) could be found as well, albeit in lower abundance. Some of these in particular show very specific spatial distributions like TG (46:3) in trachea.

The MALDI data is complementary to data of LC-MS/MS analysis in which triacylglycerides dominate. This shows the importance of combining several methods for a comprehensive picture of the lipidome in *Drosophila* larvae. While the chromatography approach reveals the high abundance of neutral TGs, MALDI suppresses these ions and unlocks the regional distribution data of phosphatidylcholines.

## Supplementary Information

Below is the link to the electronic supplementary material.

Supplementary Material 1 (PDF 3.26 MB)
