# Supplementary material for: Sample preparation of Drosophila melanogaster larvae for MALDI imaging
Source: Anal Bioanal Chem. 2026 Jan 6;418(20):6791–803. doi: 10.1007/s00216-025-06289-x (PMC13619767; doi:10.1007/s00216-025-06289-x)
Supplement: Supplementary file 1 [file 216_2025_6289_MOESM1_ESM.pdf]

Supporting Information

Sample preparation of *Drosophila melanogaster* larvae for MALDI imaging

Marius Herbst, Marie A. König, Katharina Gutbrod, Johannes Volk, Peter Dörmann, Margret H. Bülow, Marianne Engeser

Figures S1. Stainings of consecutive cryosections matching the MS spectra shown in Figure 2 in the manuscript p. 2 - 4

Figure S2. Staining of a sample dissection p. 4

Details of the staining procedure p. 4

Details for the MS analysis: segmentation and boxplot creation p. 5

Figures S3 – S6. Segmentation and boxplots for sample dissection No 1 shown in the manuscript p. 5 - 8

Figure S7 – S11. Segmentation and boxplots for sample dissection No 2 p. 9 - 12

Figure S12 – S16. Segmentation and boxplots for sample dissection No 3 p. 13 - 16

Figure S17. Segmentation for sample dissection No 4 for which the washing step with ddH_2O was omitted p. 17

Supporting Figure S1. Brightfield, phalloidin-TRITC (shown in red, middle panel) and BODIPY 493/503 (shown in green, right panel) stainings of consecutive cryosections matching MS spectra shown in Figure 2. Scale bars represent 500 µm.

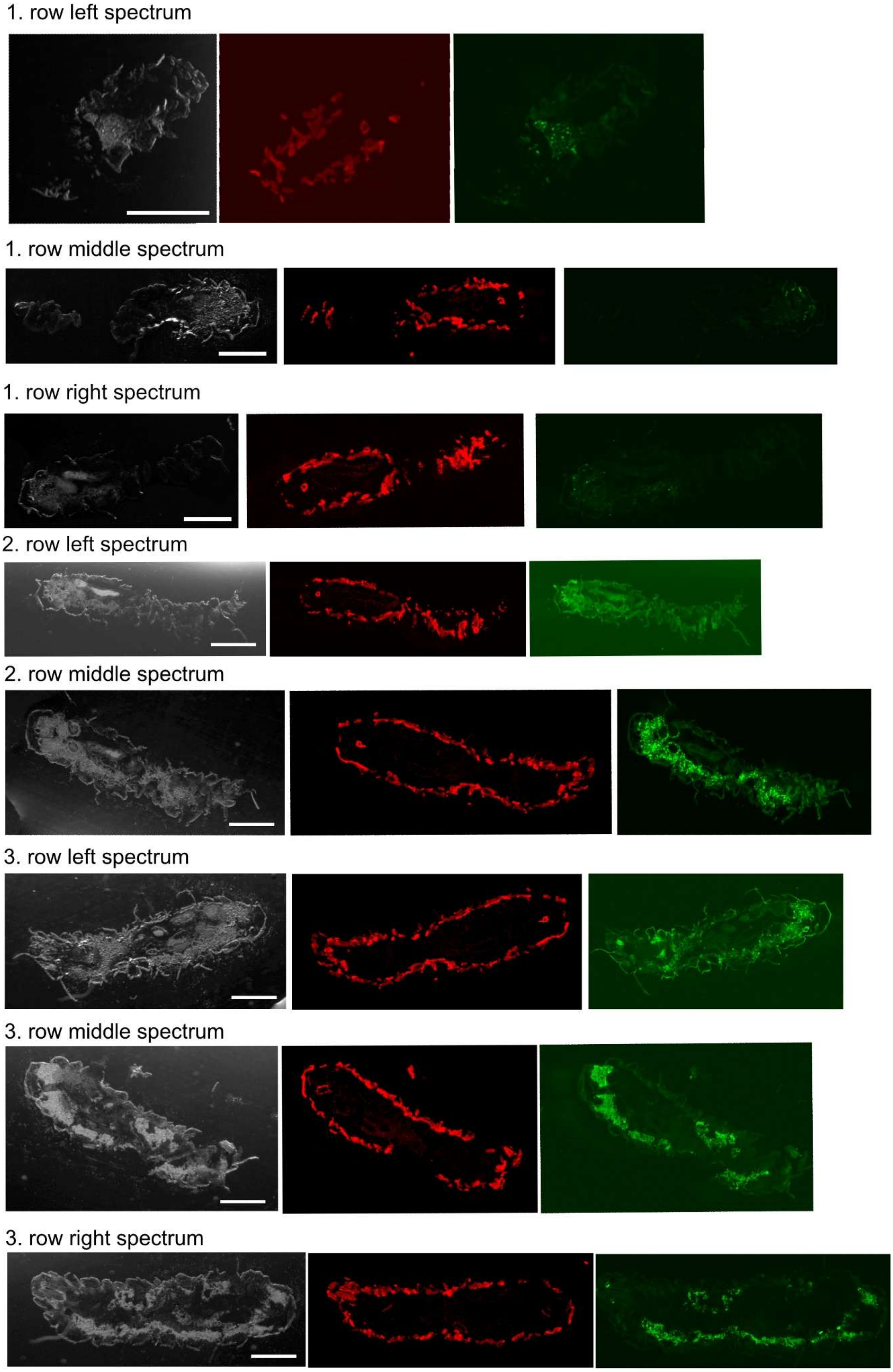

4. row left spectrum

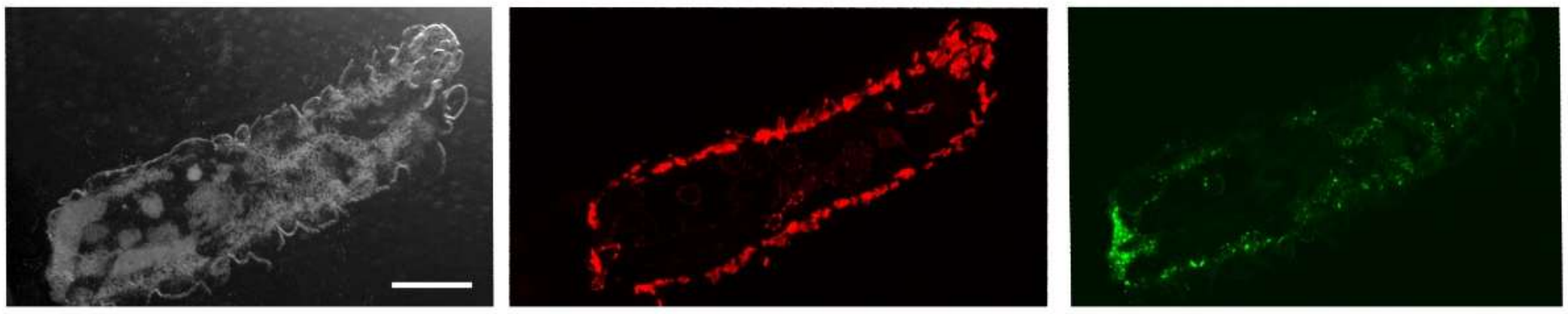

4. row middle spectrum

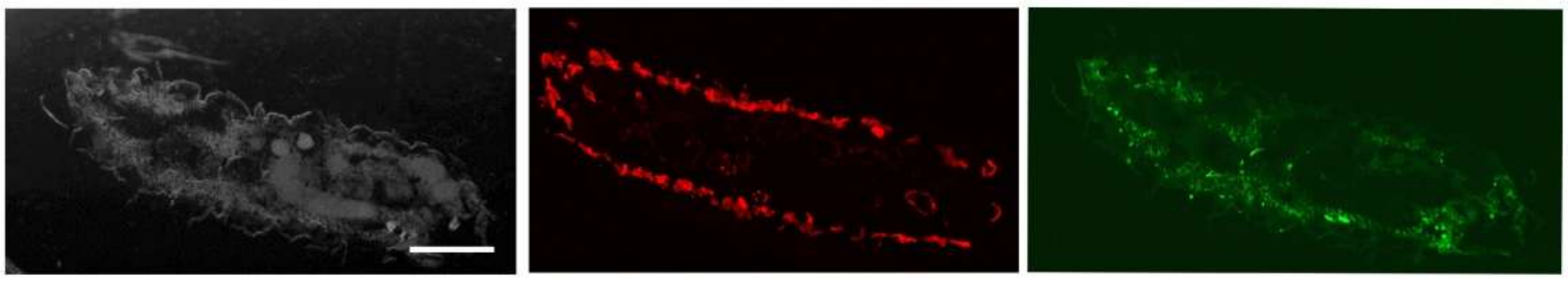

4. row right spectrum

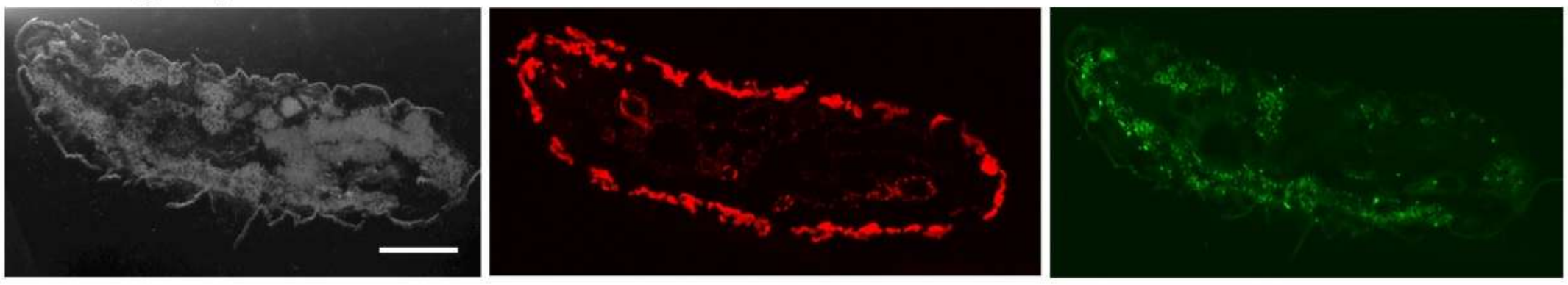

5. row left spectrum

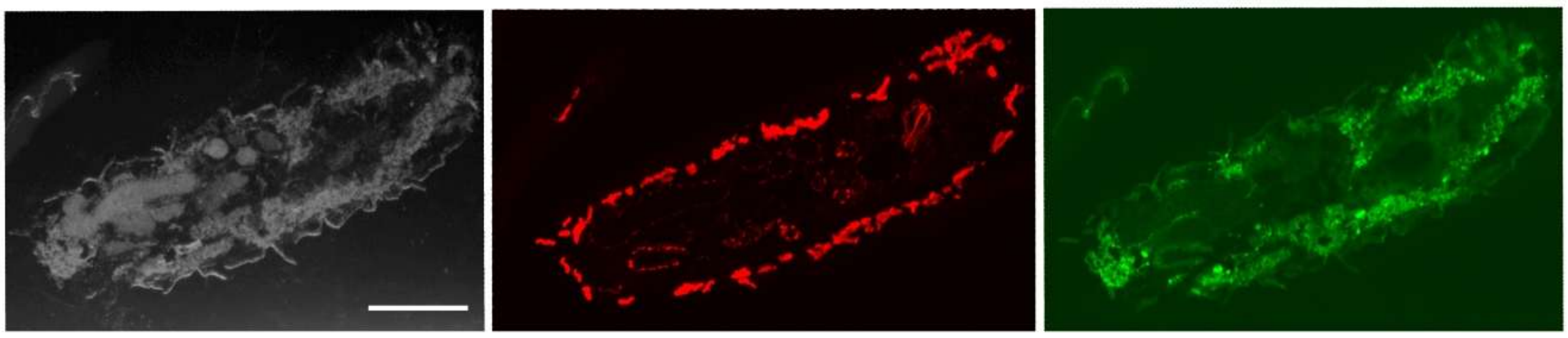

5. row middle spectrum

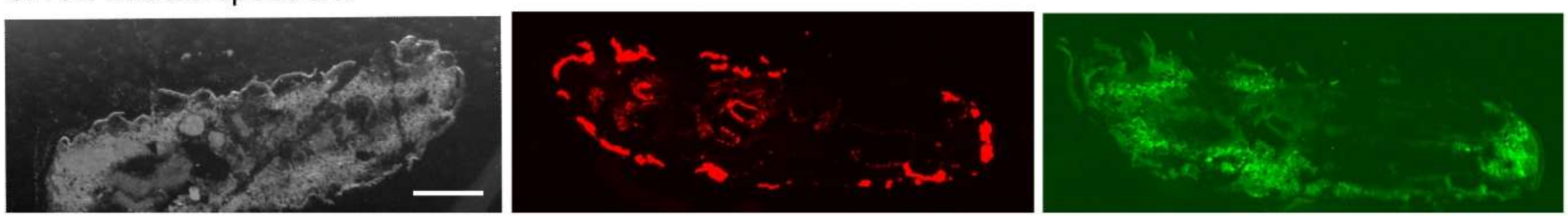

5. row right spectrum

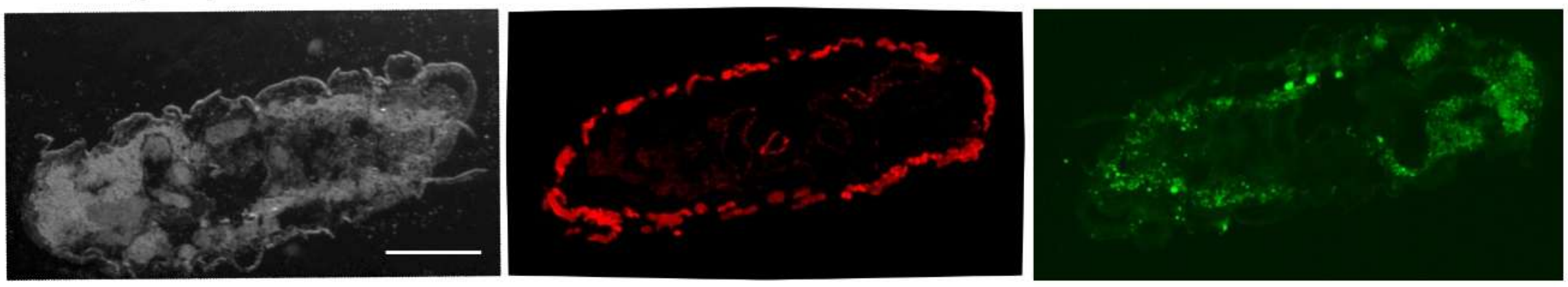

6. row left spectrum

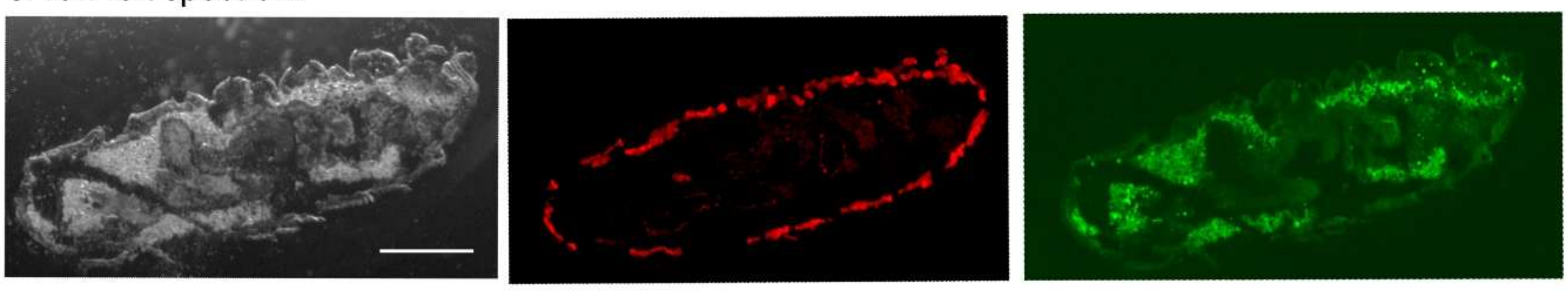

6. row middle spectrum

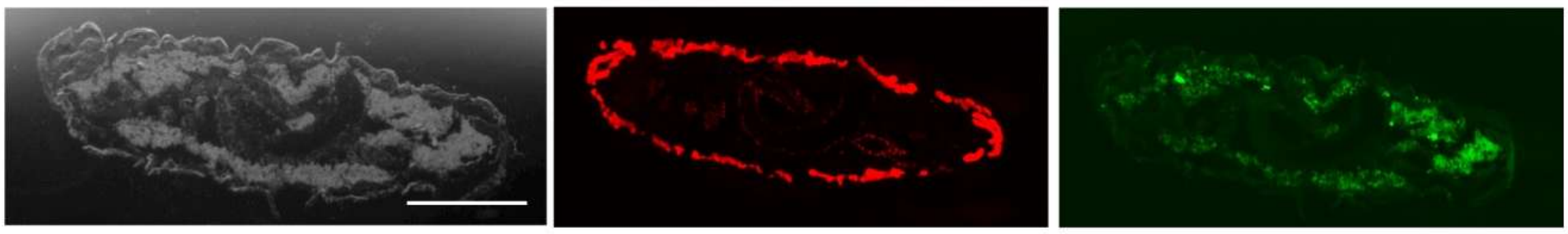

6. row right spectrum

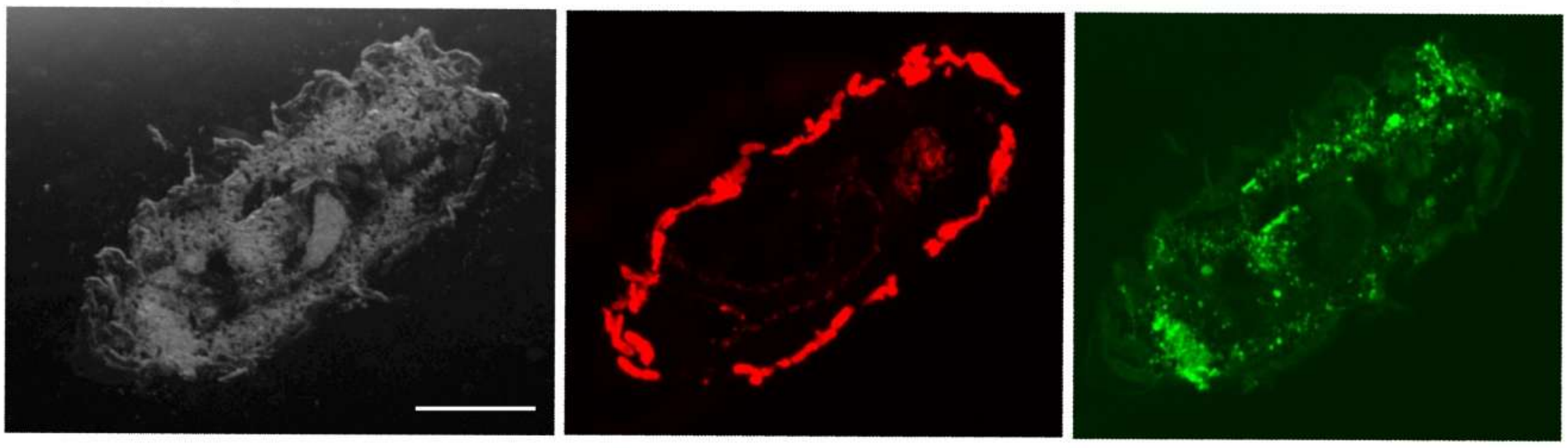

Supporting Figure S2. Drosophila *larval organs after dissection. A) Brightfield image shows inverted cuticle with bodywall muscles that was excluded from MS analysis due to sample thickness. Gut, brain, trachea and fat body are identifiable based on morphology. B) phalloidin-TRITC staining labelling F-actin shows the muscle-rich bodywall and muscle-rich gut regions. C) BODIPY 493/503 staining labels lipid droplets in fat body, gut and brain. Scale bars represent 200 µm.*

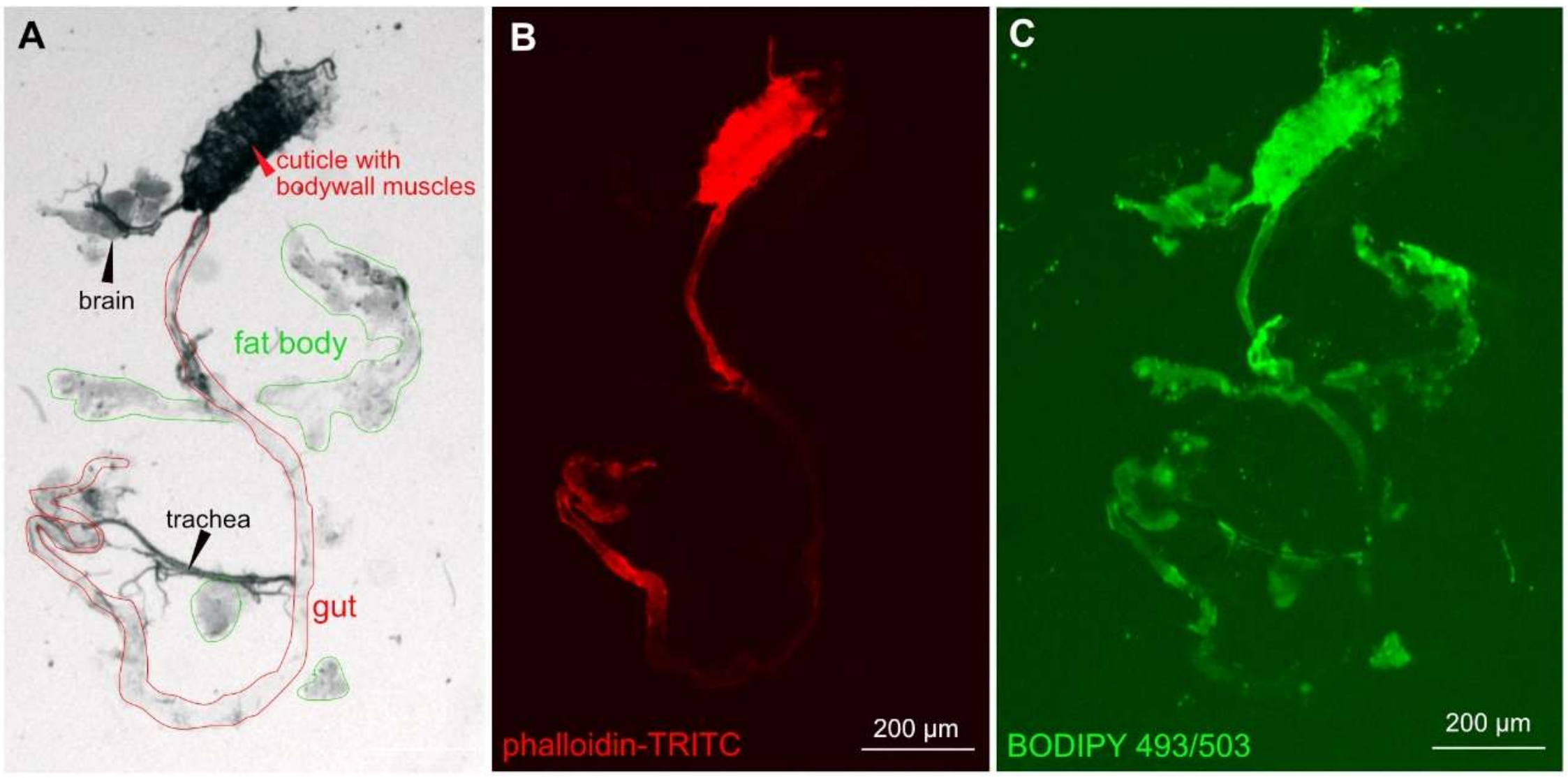

Staining procedure

Dissected tissue was incubated in phalloidin-TRITC and BODIPY 493/503 (both 1 µM) on a microscope slide in a drop of PBS for 15 minutes. Tissue was carefully washed 3 times in PBS before imaging with a NIKON SMZ-25 stereomicroscope with epifluorescence. Likewise, cryosections were incubated on a microscope slide with phalloidin-TRITC and BODIPY 493/503 (both 1 µM) for 15 min, washed and imaged immediately.

Segmentation and Intensity Boxplots

Segmentation analysis was performed utilizing the Segmentation Pipeline of the SCiLS Lab software (Bruker Daltonik). The features of the measurement are found by orthogonal matching pursuit. Per spectrum hundred peaks are selected and this is done for every 16th spectrum. Using the mean spectrum of the respective measurement, the resulting peaks are moved to the local maximum. Segmentation is then performed with bisecting k-means considering also the correlation distance and medium denoising. The resulting clusters where then declared as subregions and their respective mean spectra were calculated. Afterwards the identified *m/z*-values of the detected lipids from the whole measurement region were co-localized to the subregions and the five most characteristic ions for each region were chosen and their abundance displayed through ion intensity boxplots.

Segmentation for Dissection No 1

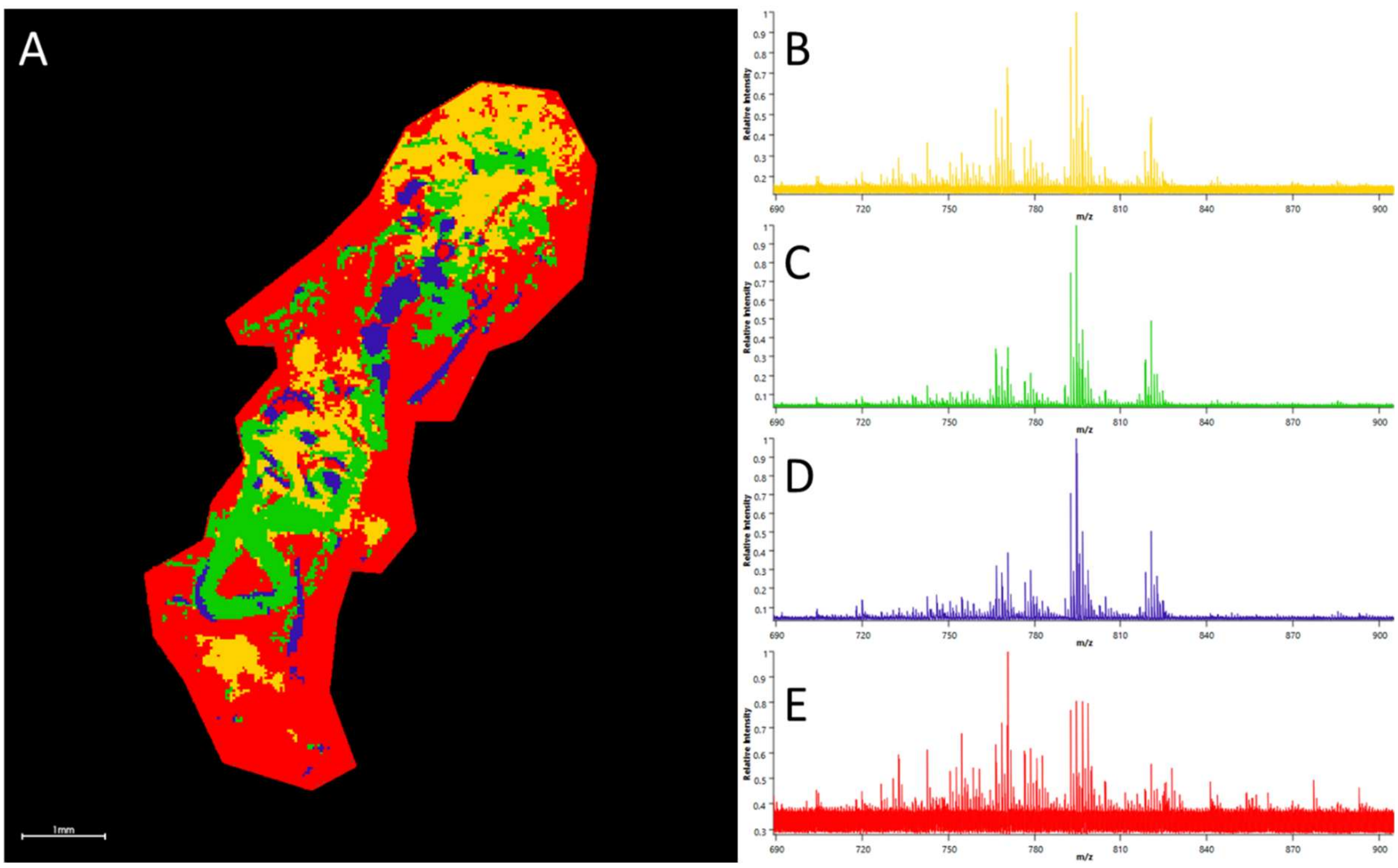

Supporting Figure S3. A) Segmentation clusters as seen in Fig. 4b. B) Mean spectrum of yellow cluster corresponding to fatbody region. C) Mean Spectrum of green cluster corresponding to gut region. D) Mean spectrum of blue cluster corresponding to trachea/other region. E) Mean spectrum of red cluster corresponding to offsample region.

m/z-values co-localized to fatbody region

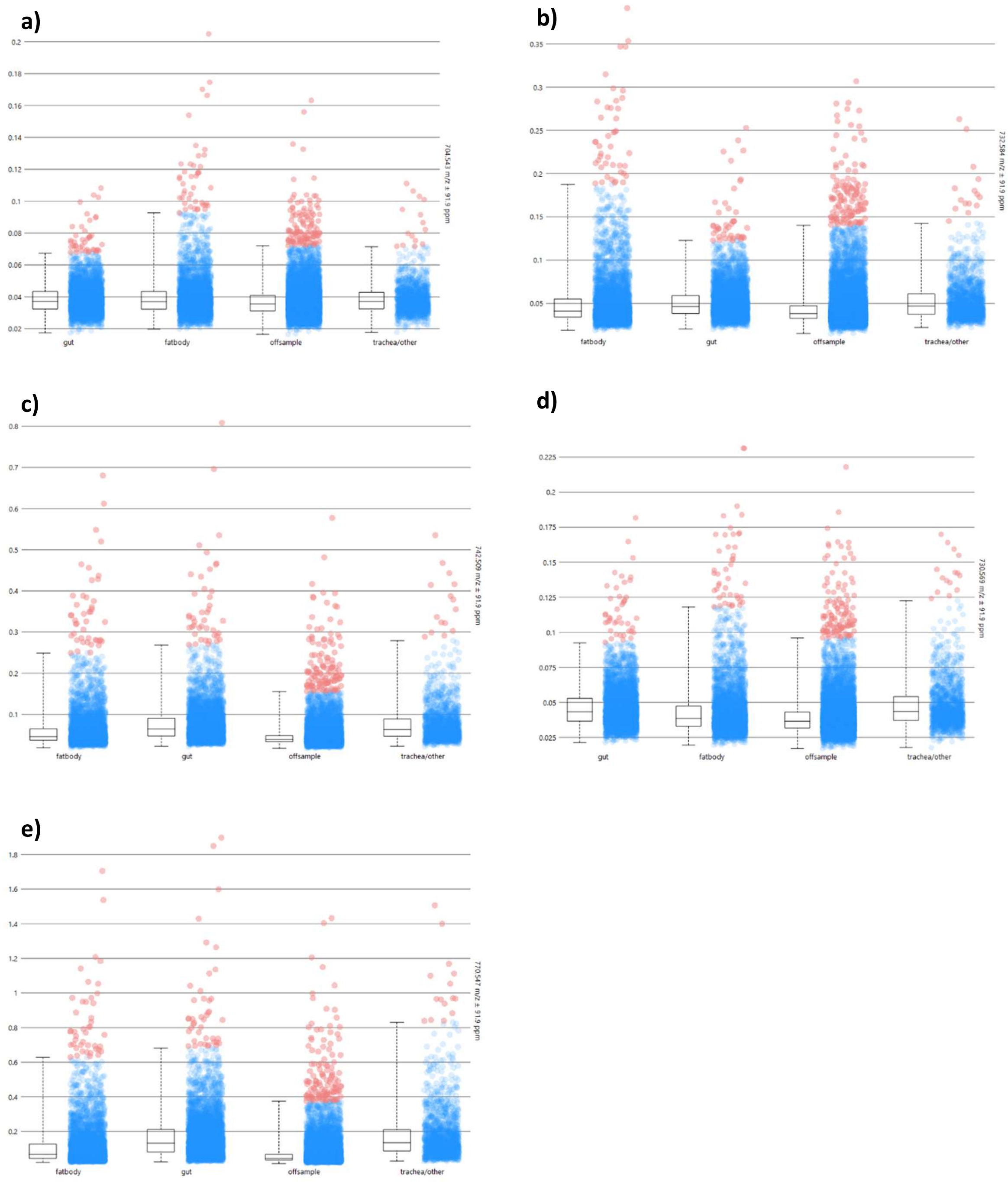

Supporting Figure S4. Intensity box plots for different sample regions shown in Fig. S3 of a) m/z 704.543; b) m/z 732.584; c) m/z 742.509; d) m/z 730.569; e) m/z 770.547 co-localized to fatbody region.

m/z-values co-localized to gut region

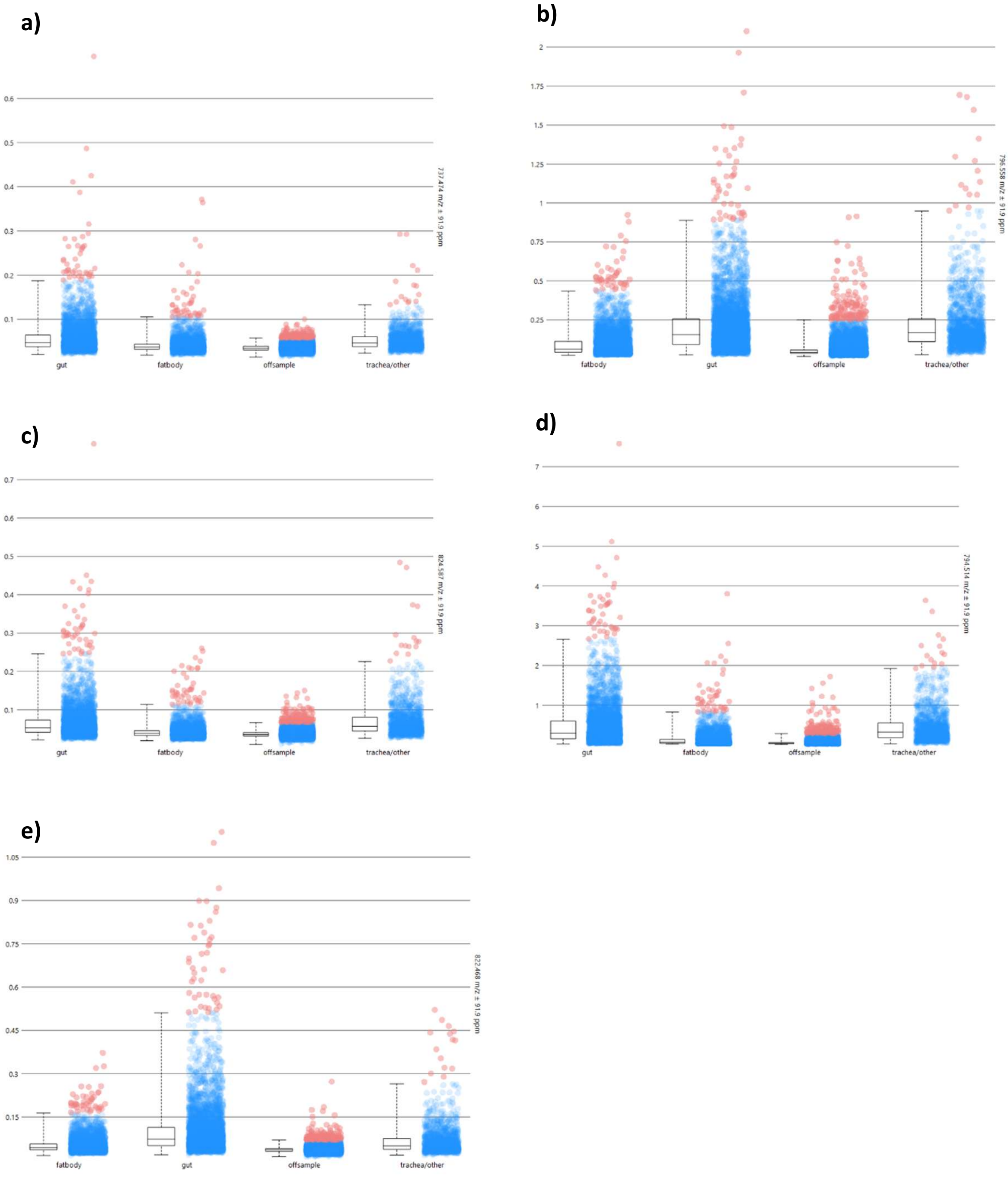

Supporting Figure S5. Intensity box plots for different sample regions shown in Fig. S3 of a) m/z 737.474; b) m/z 796.558; c) m/z 824.587; d) m/z 794.514; e) m/z 822.468 co-localized to gut region.

m/z-values co-localized to trachea/other region

a)

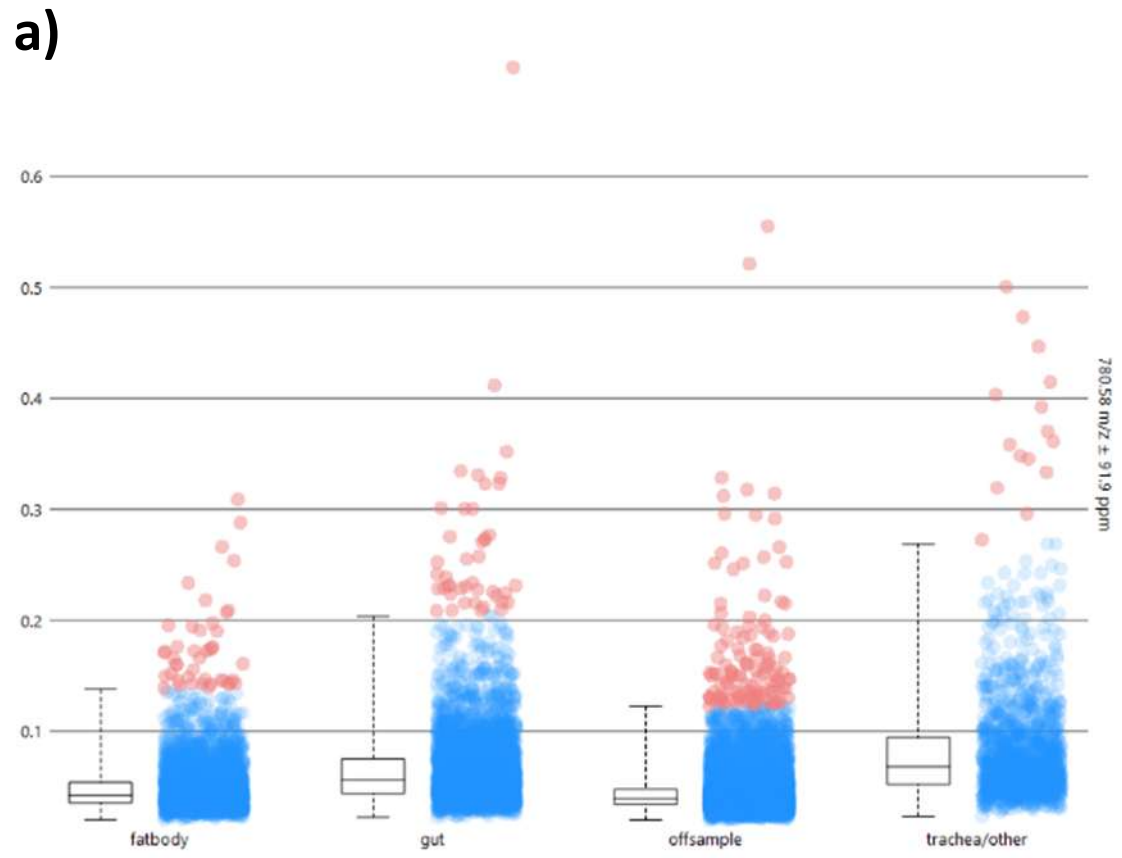

b)

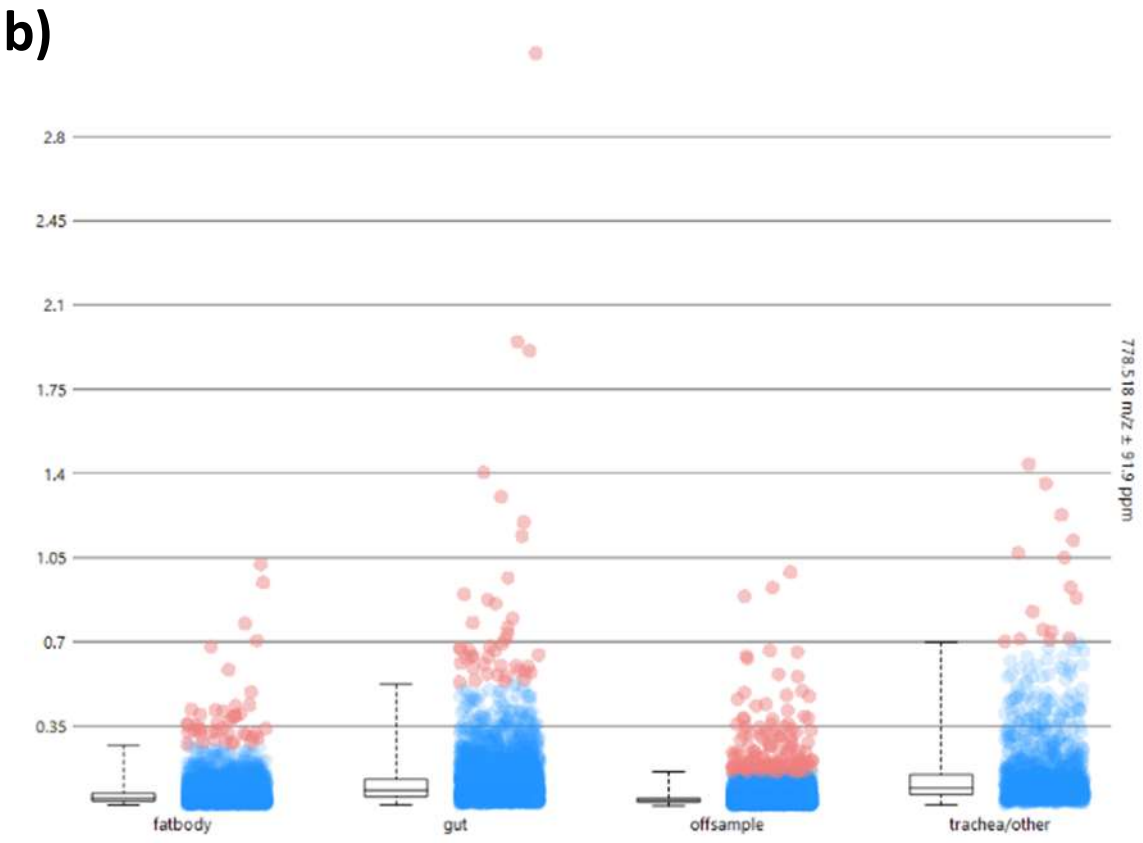

c)

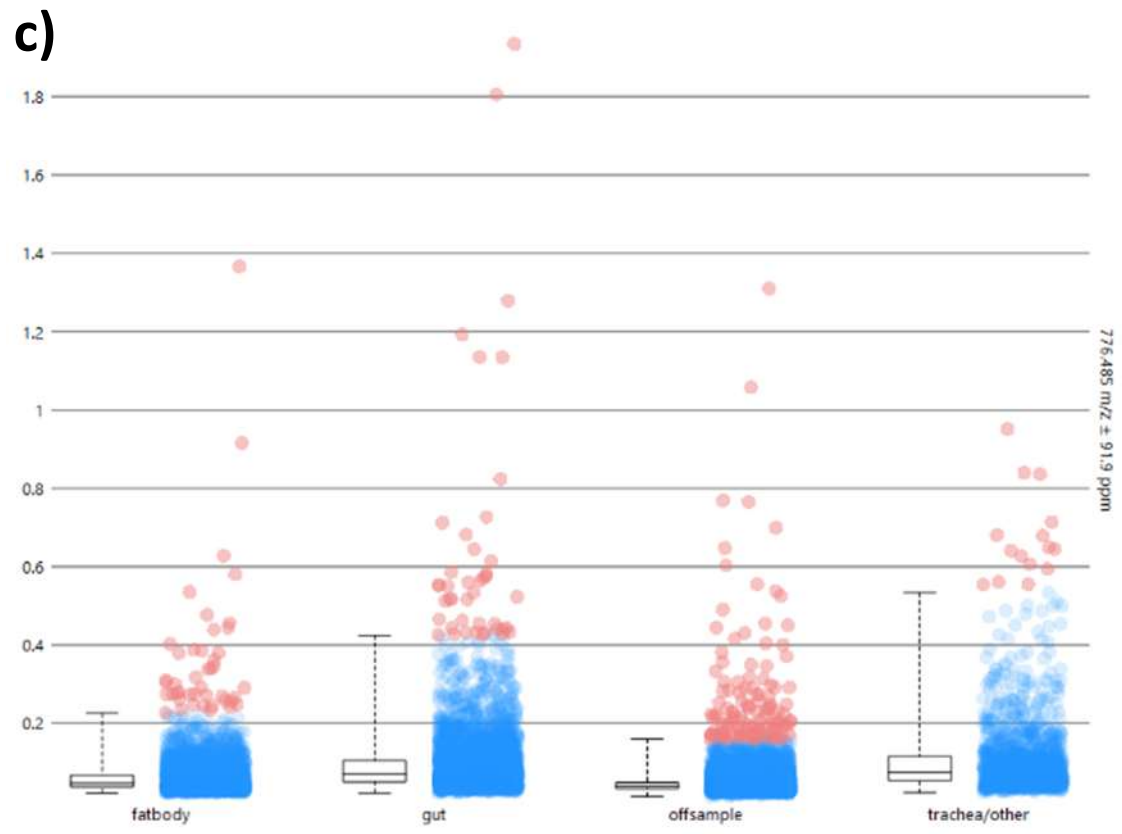

d)

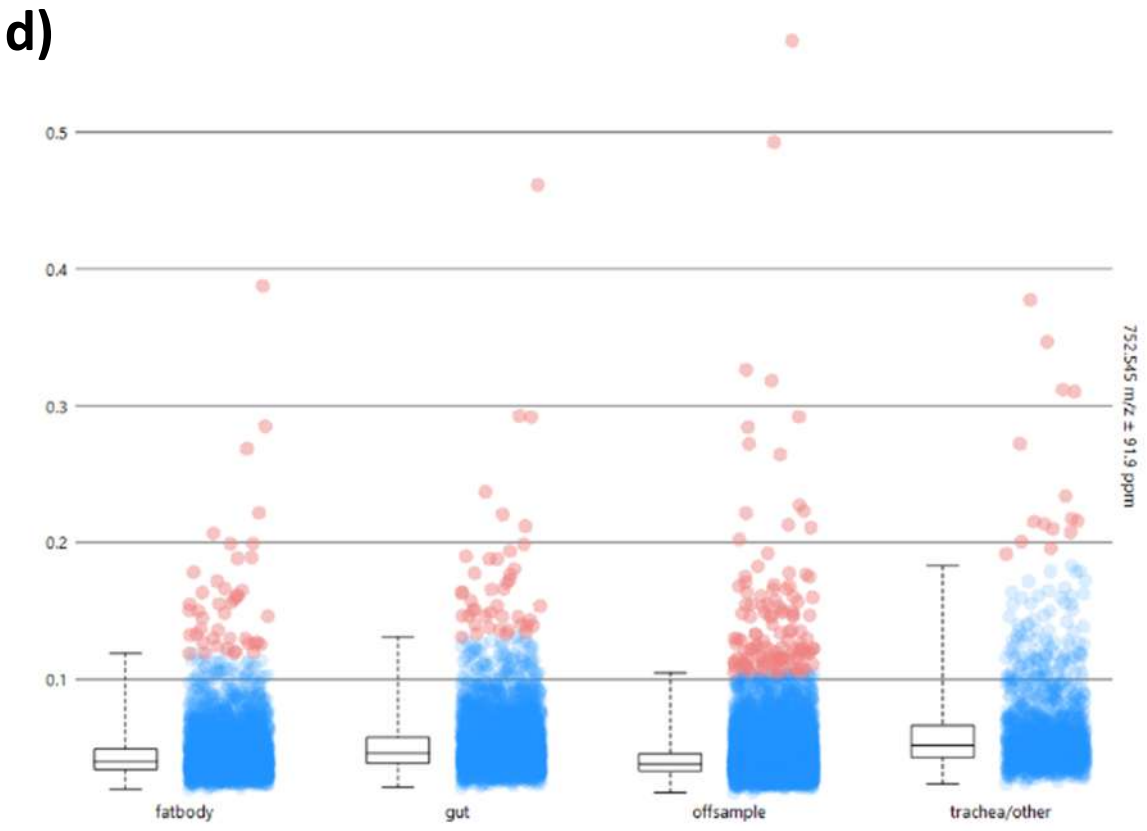

e)

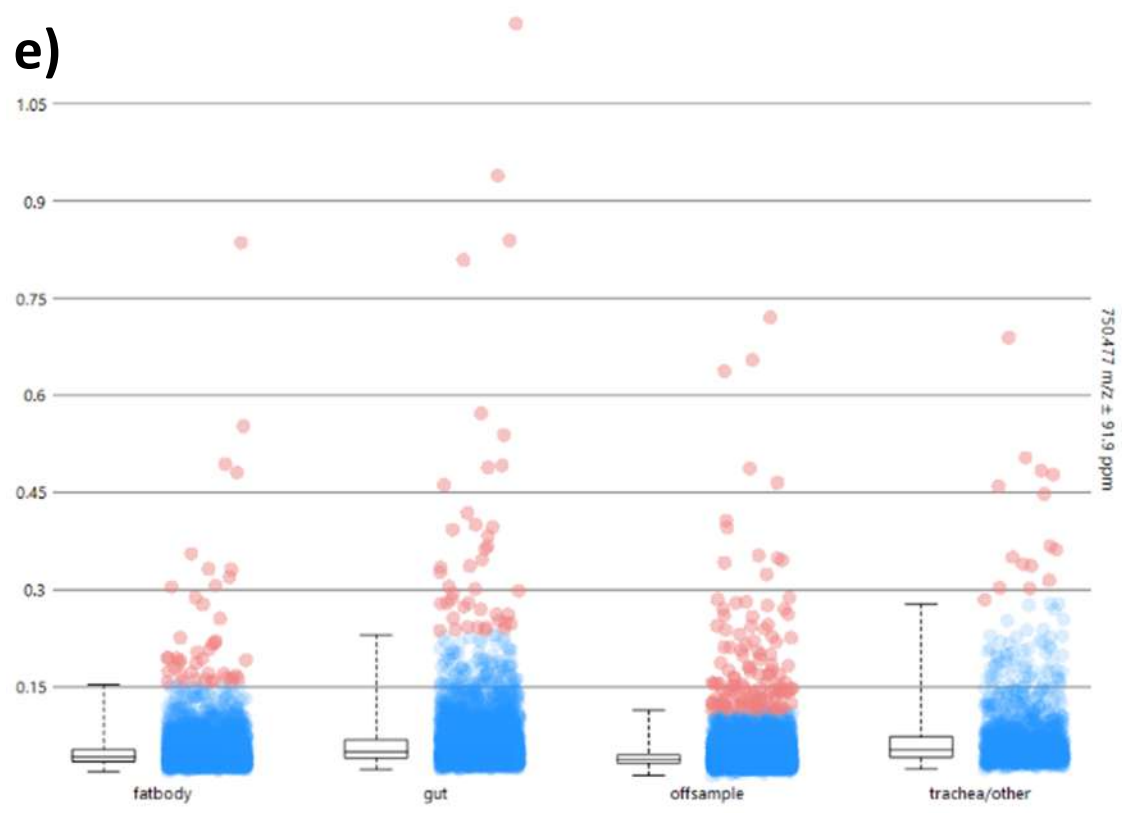

f)

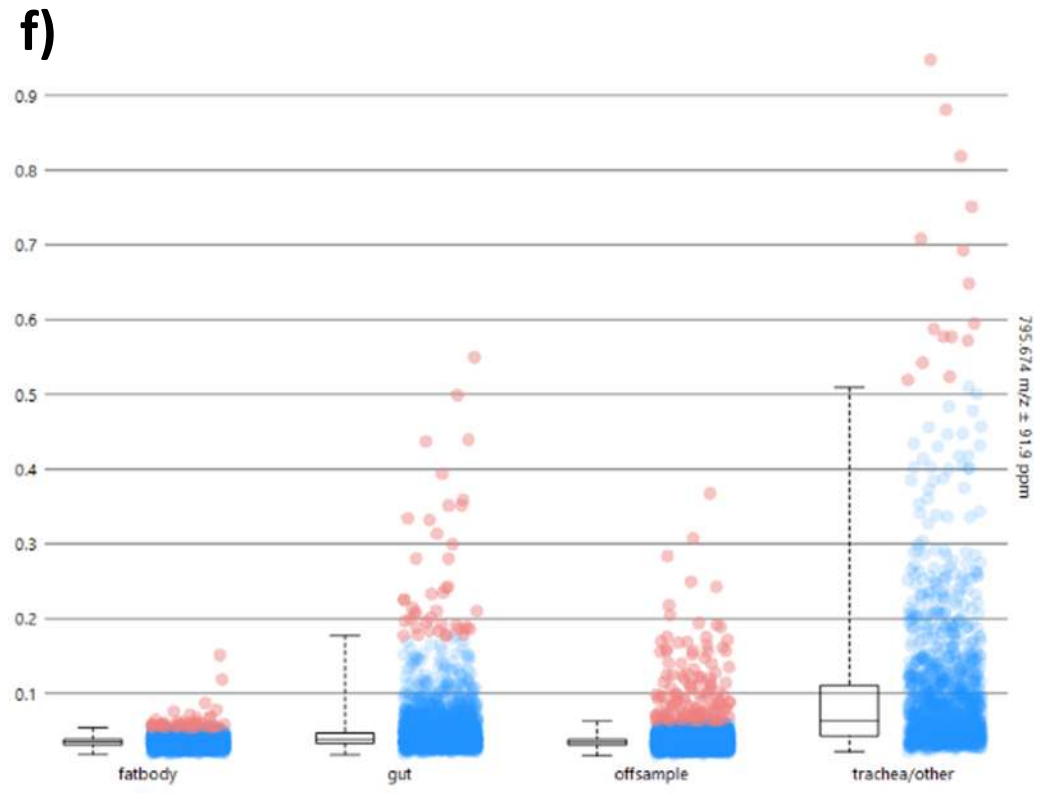

g)

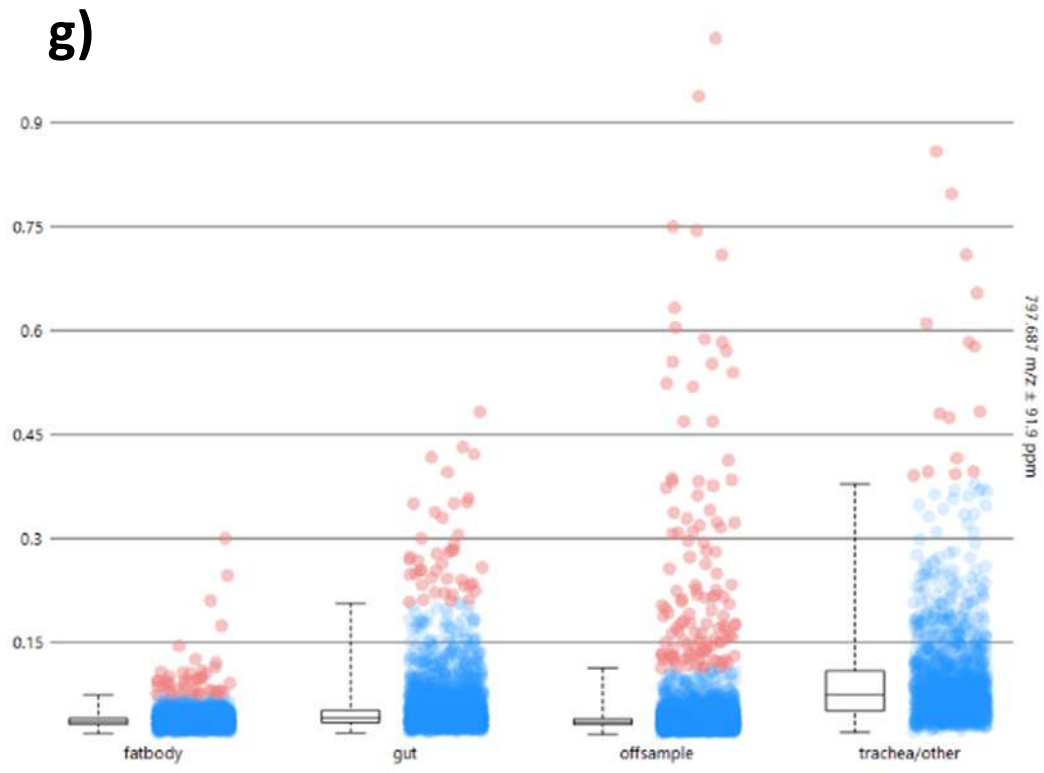

Supporting Figure S6. Intensity box plots for different sample regions shown in Fig. S3 of a) m/z 780.58; b) m/z 778.518; c) m/z 776.485; d) m/z 752.545; e) m/z 750.477, f) 795.674; g) 797.687 co-localized to trachea/other region.

Segmentation for Dissection No 2

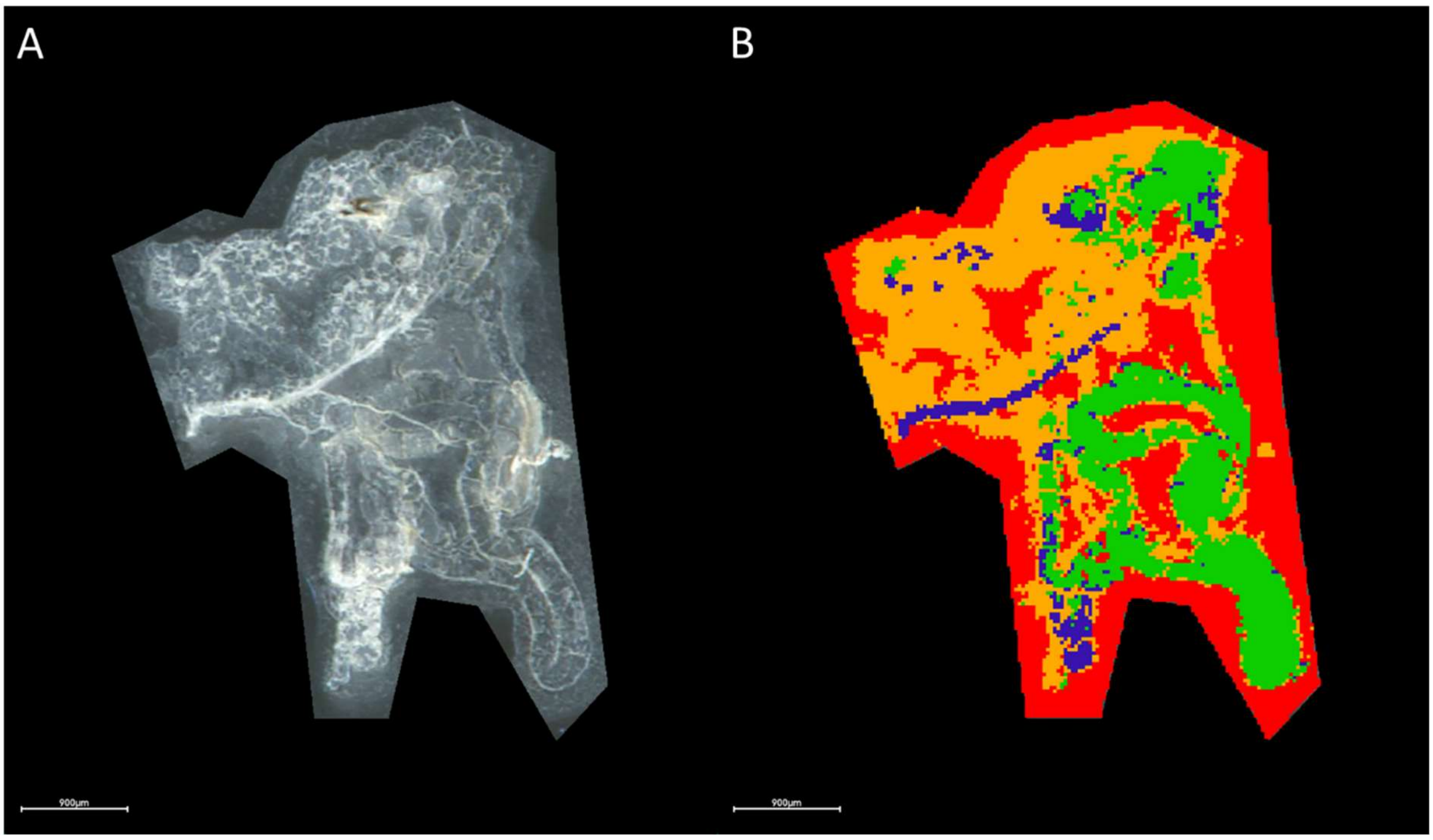

Supporting Figure S7. A) Optical image of dissected Drosophila *larva. B) Clusters from Segmentation Pipeline showing four different regions: offsample (red), gut (green), fatbody (yellow) and trachea/other (blue).*

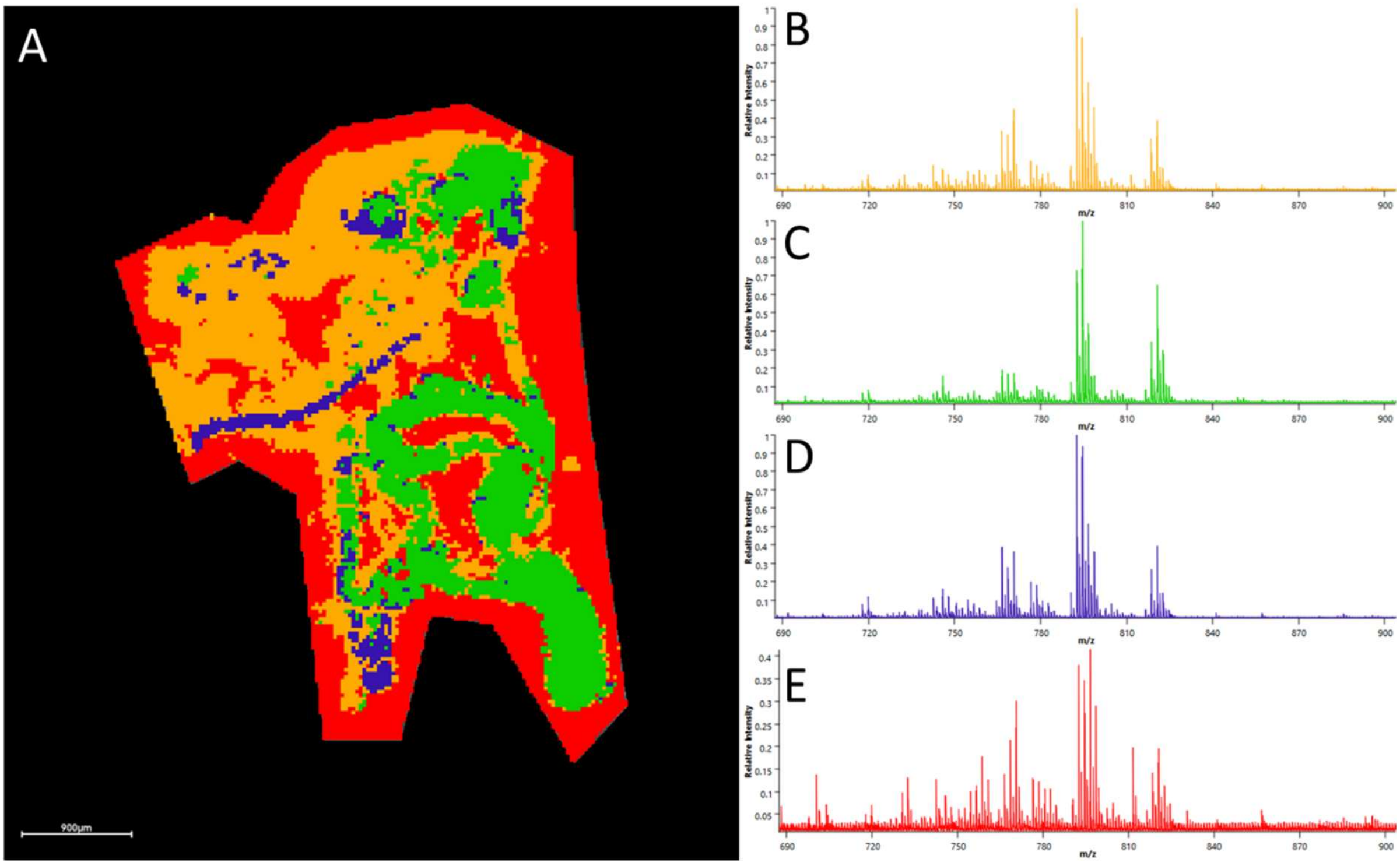

Supporting Figure S8. A) Segmentation clusters as seen in Supp. Fig. S7B. B) Mean spectrum of yellow cluster corresponding to fatbody region. C) Mean Spectrum of green cluster corresponding to gut region. D) Mean spectrum of blue cluster corresponding to trachea/other region. E) Mean spectrum of red cluster corresponding to offsample region.

m/z-values co-localized to fatbody region

a)

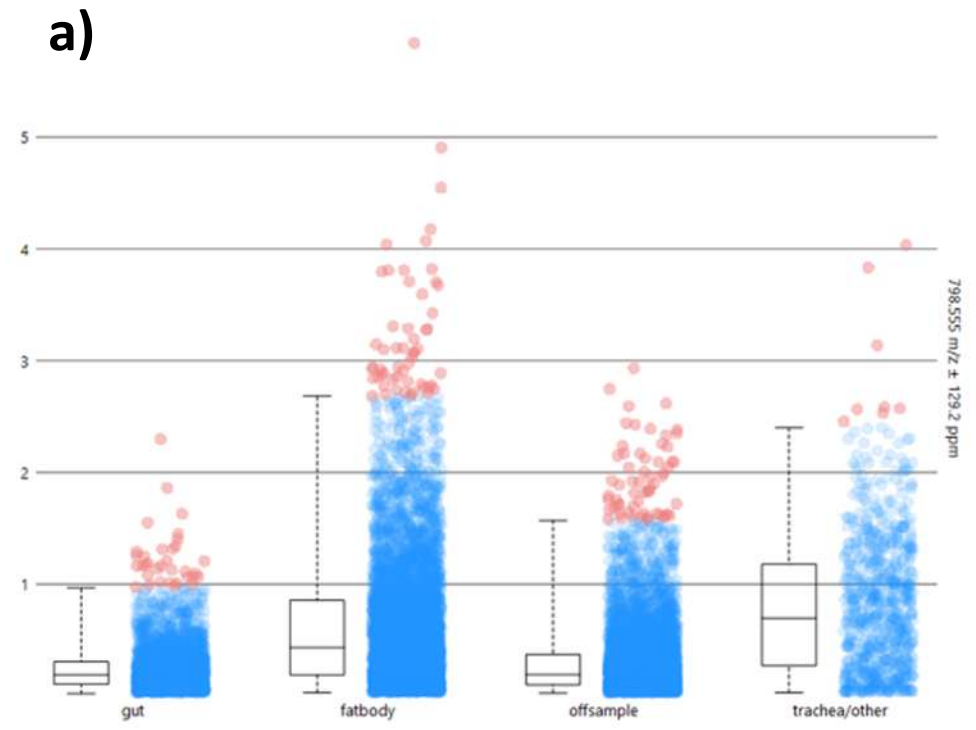

b)

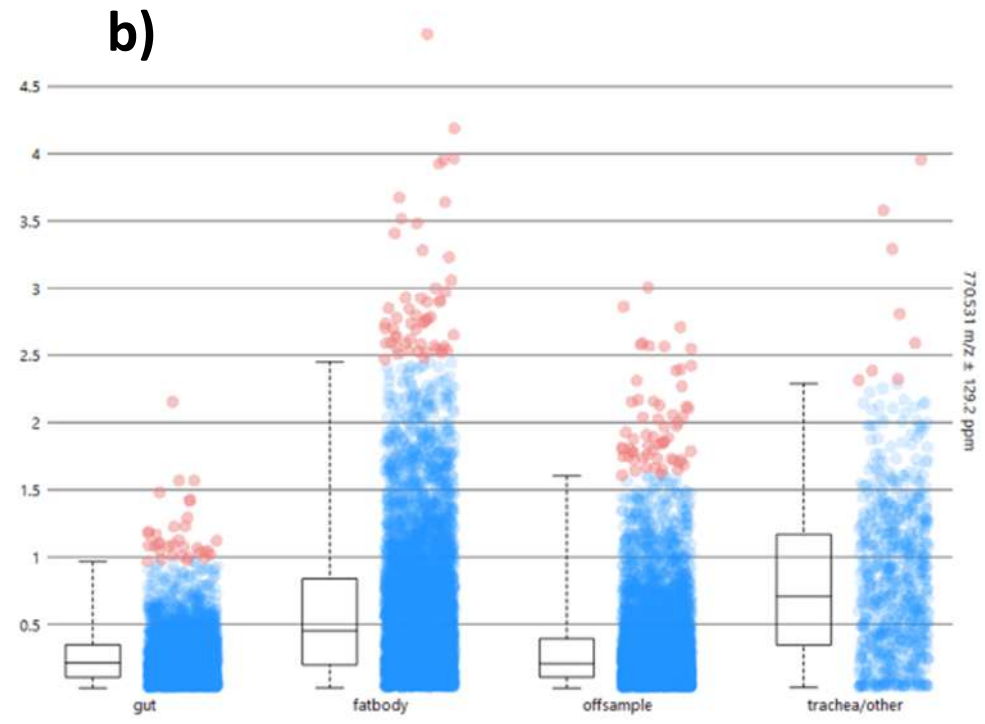

c)

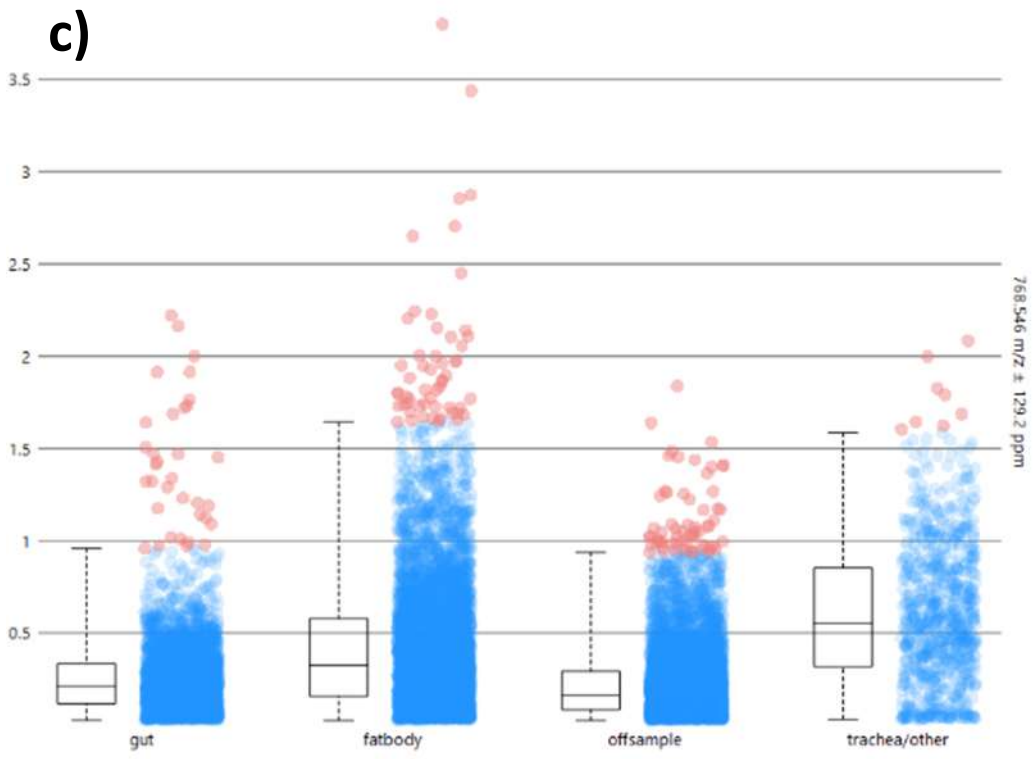

d)

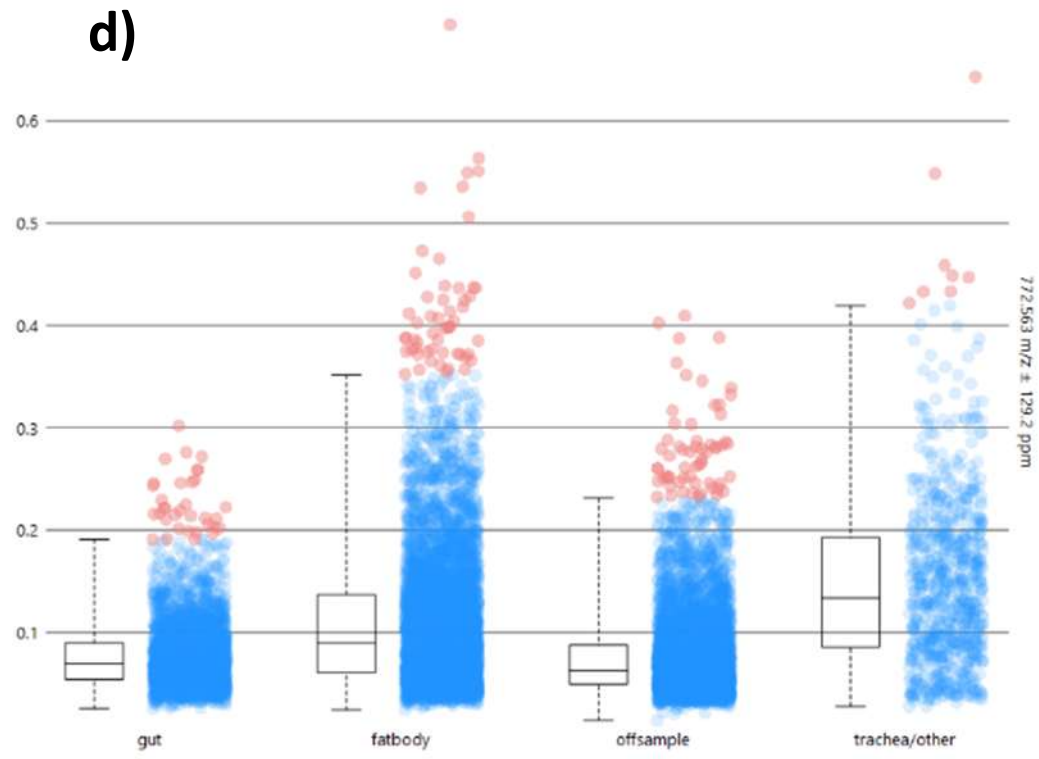

e)

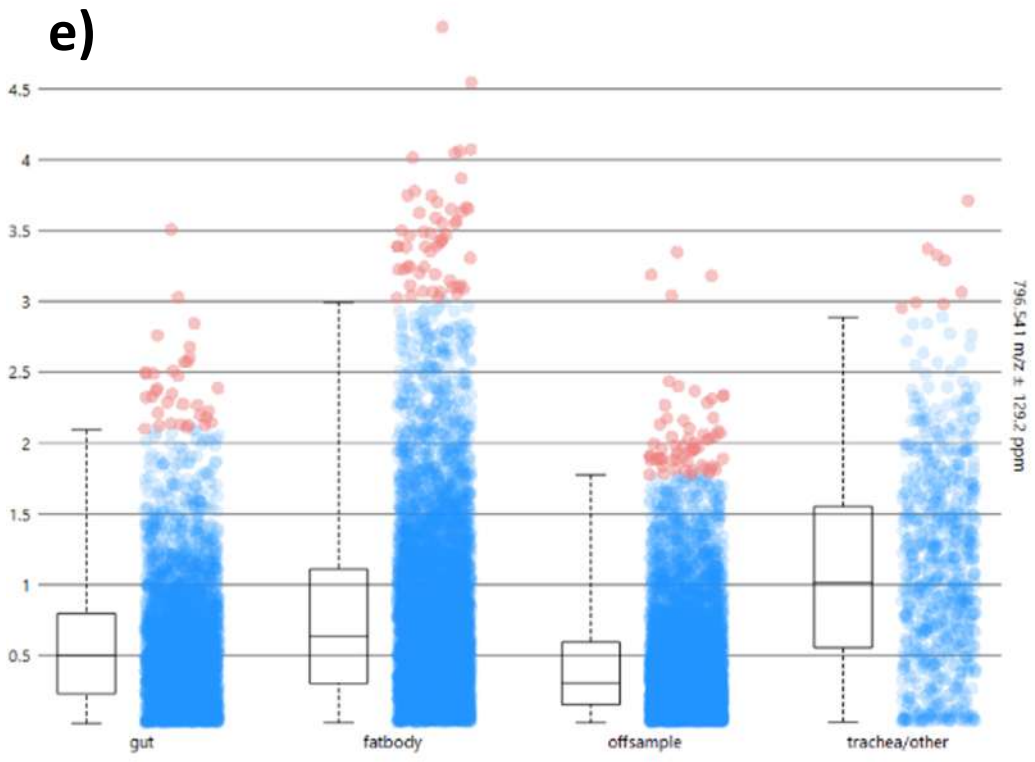

Supporting Figure S9. Intensity box plots for different sample regions shown in Fig. S7 of a) m/z 798.555; b) m/z 770.531; c) m/z 768.546; d) m/z 772.563; e) m/z 796.541 co-localized to fatbody region.

m/z-values co-localized to gut region

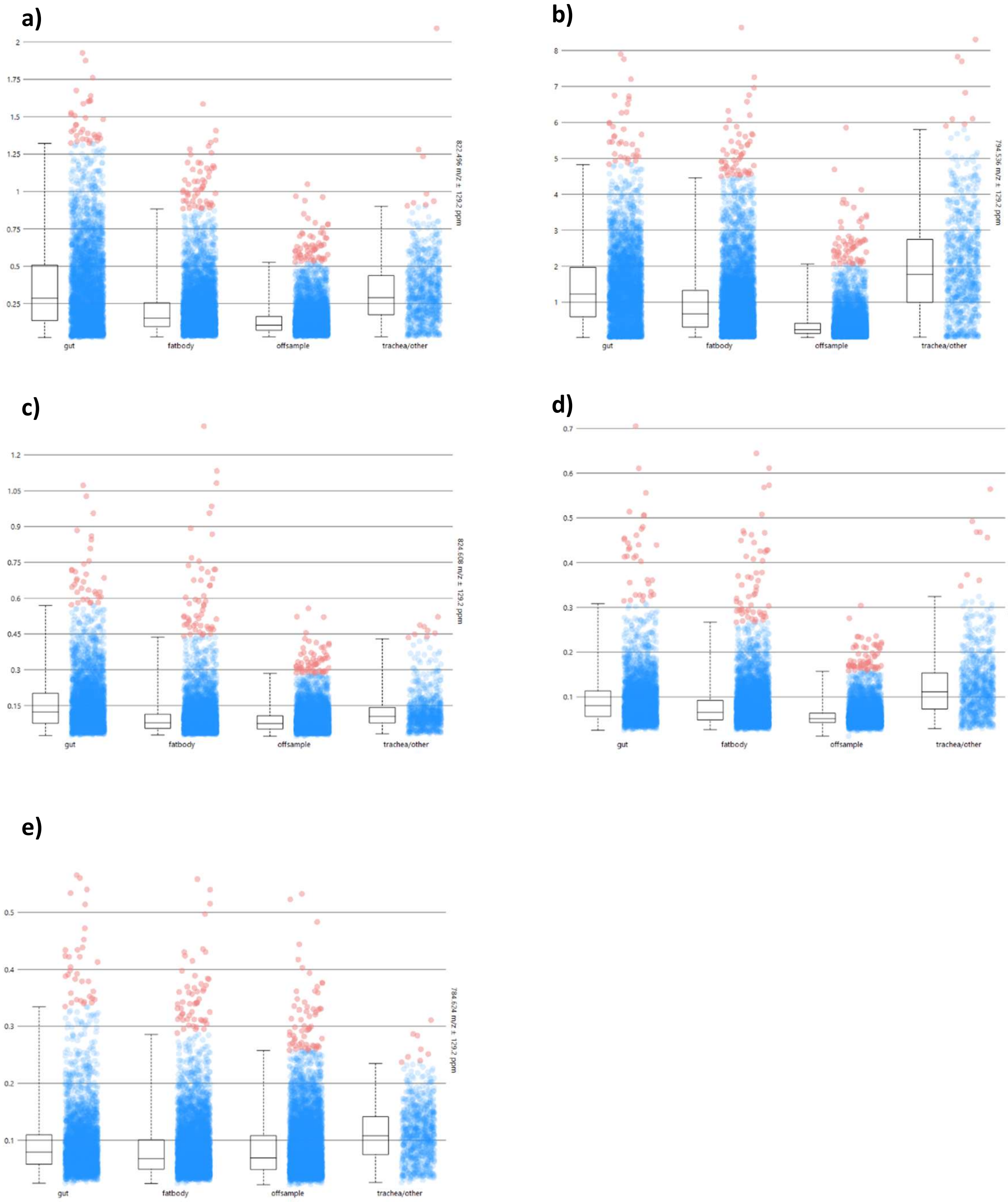

Supporting Figure S10. Intensity box plots for different sample regions shown in Fig. S7 of a) m/z 822.496; b) m/z 794.536; c) m/z 824.608; d) m/z 737.503; e) m/z 784.624 co-localized to gut region.

m/z-values co-localized to trachea/other region

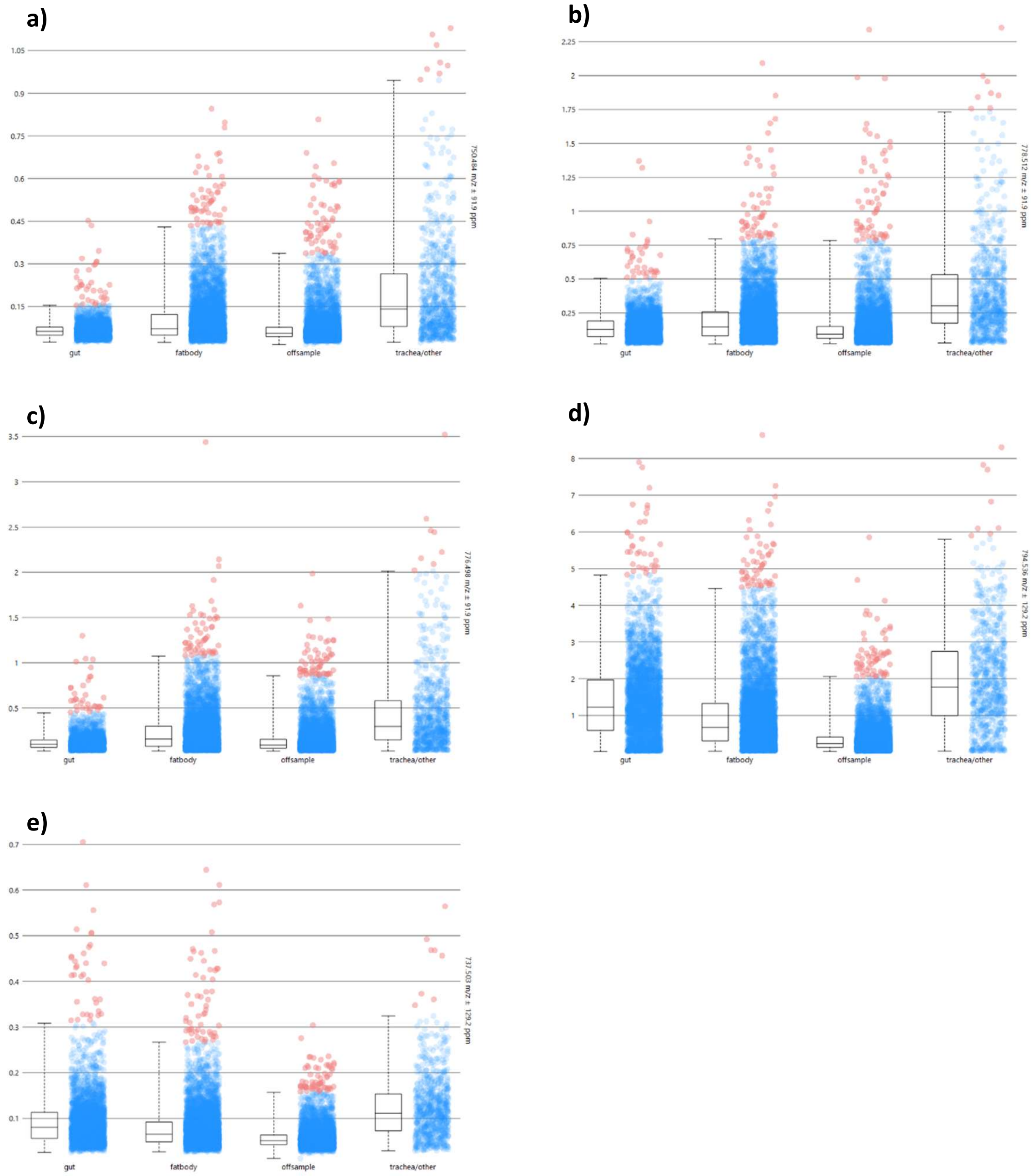

Supporting Figure S11. Intensity box plots for different sample regions shown in Fig. S7 of a) m/z 750.484; b) m/z 778.512; c) m/z 776.498; d) m/z 794.536; e) m/z 737.503 co-localized to trachea/other region.

Segmentation for Dissection No 3

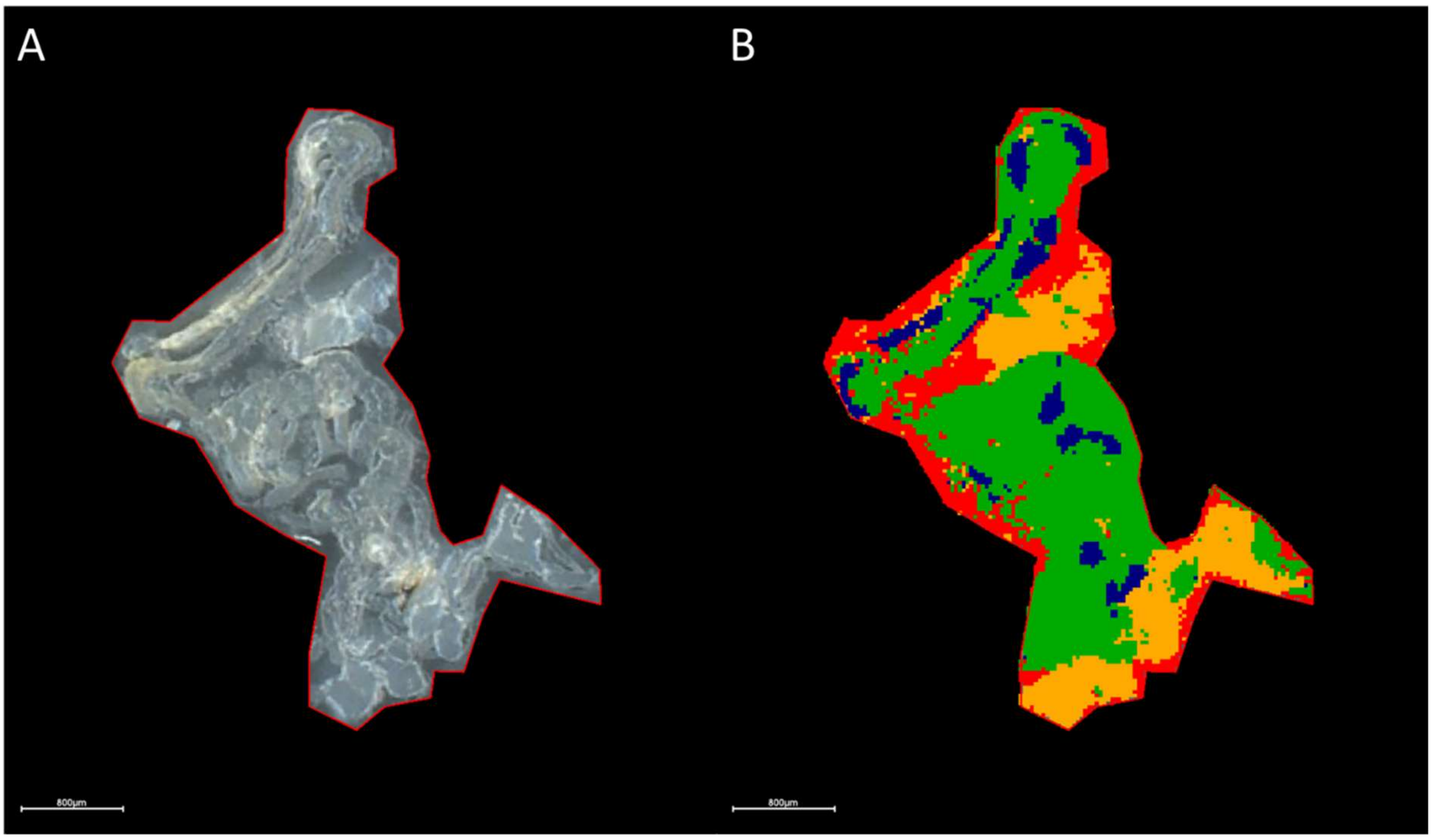

Supporting Figure S12. A) Optical image of dissected Drosophila *larva. B) Clusters from Segmentation Pipeline showing four different regions: offsample (red), gut (green), fatbody (yellow) and trachea/other (blue).*

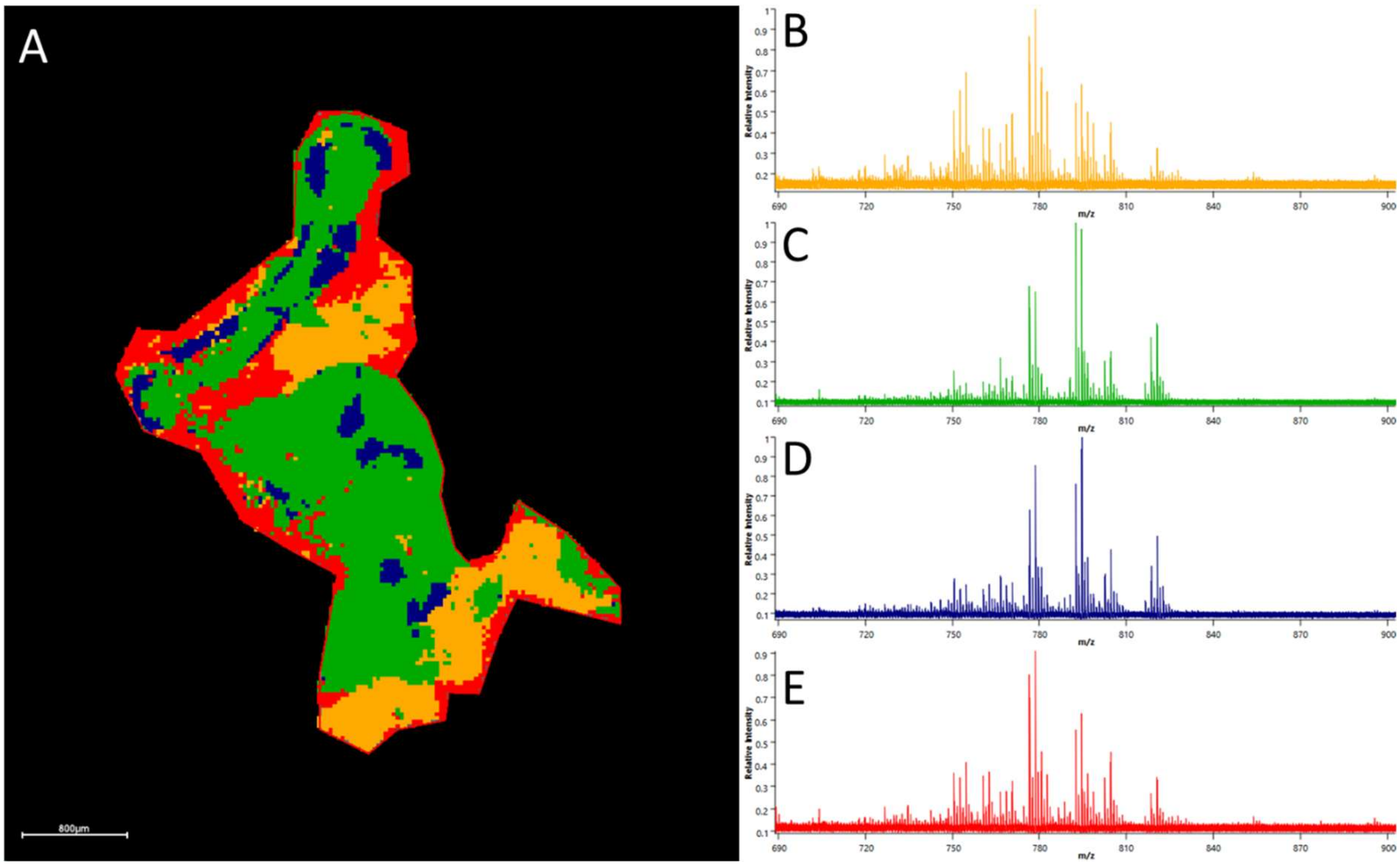

Supporting Figure S13. A) Segmentation clusters as seen in Supp. Fig. S12B. B) Mean spectrum of yellow cluster corresponding to fatbody region. C) Mean Spectrum of green cluster corresponding to gut region. D) Mean spectrum of blue cluster corresponding to trachea/other region. E) Mean spectrum of red cluster corresponding to offsample region.

m/z-values co-localized to fatbody region

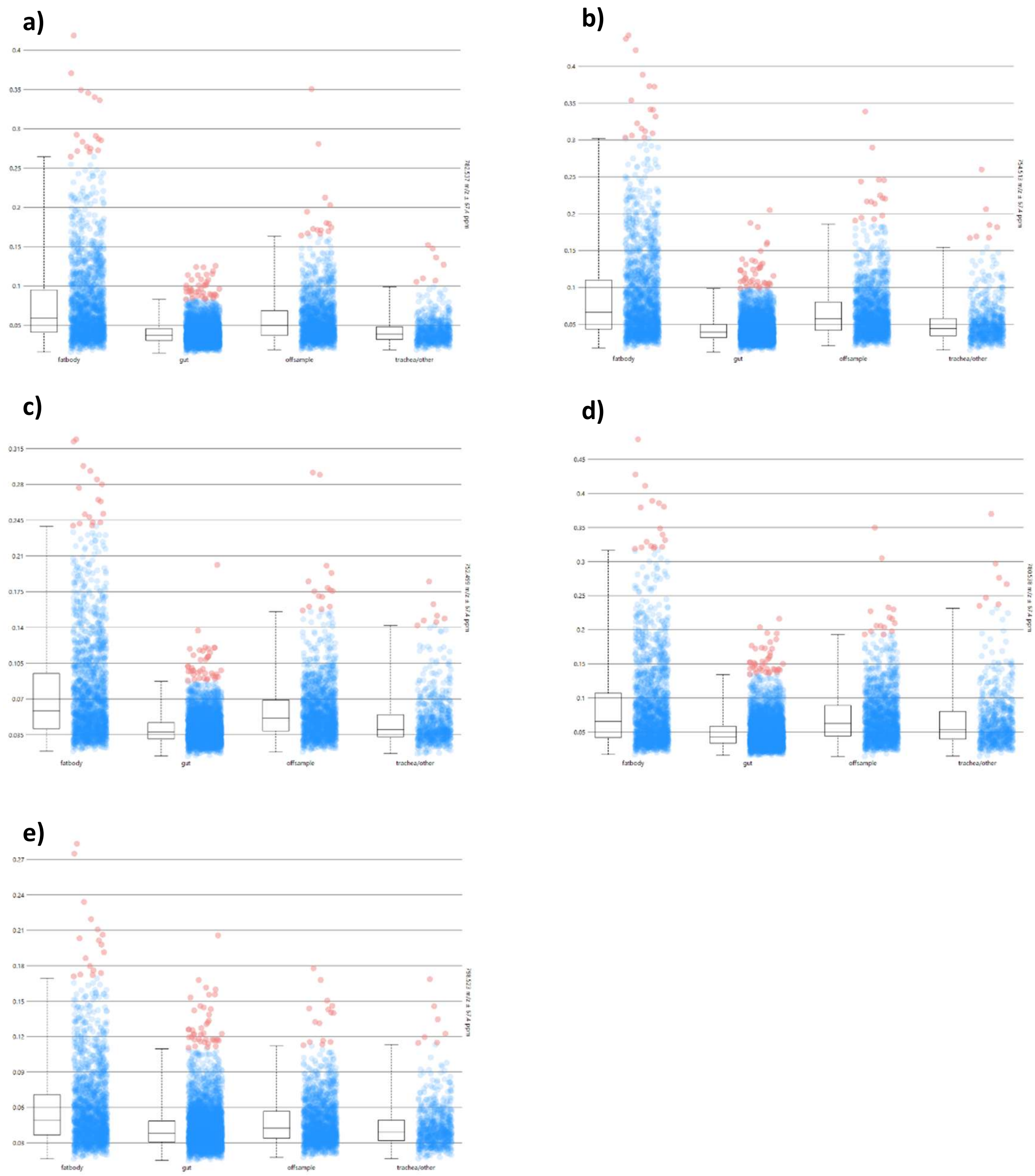

Supporting Figure S14. Intensity box plots for different sample regions shown in Fig. S12 of a) m/z 782.593; b) m/z 754.559; c) m/z 752.557; d) m/z 780.573; e) m/z 798.573 co-localized to fatbody region.

m/z-values co-localized to gut region

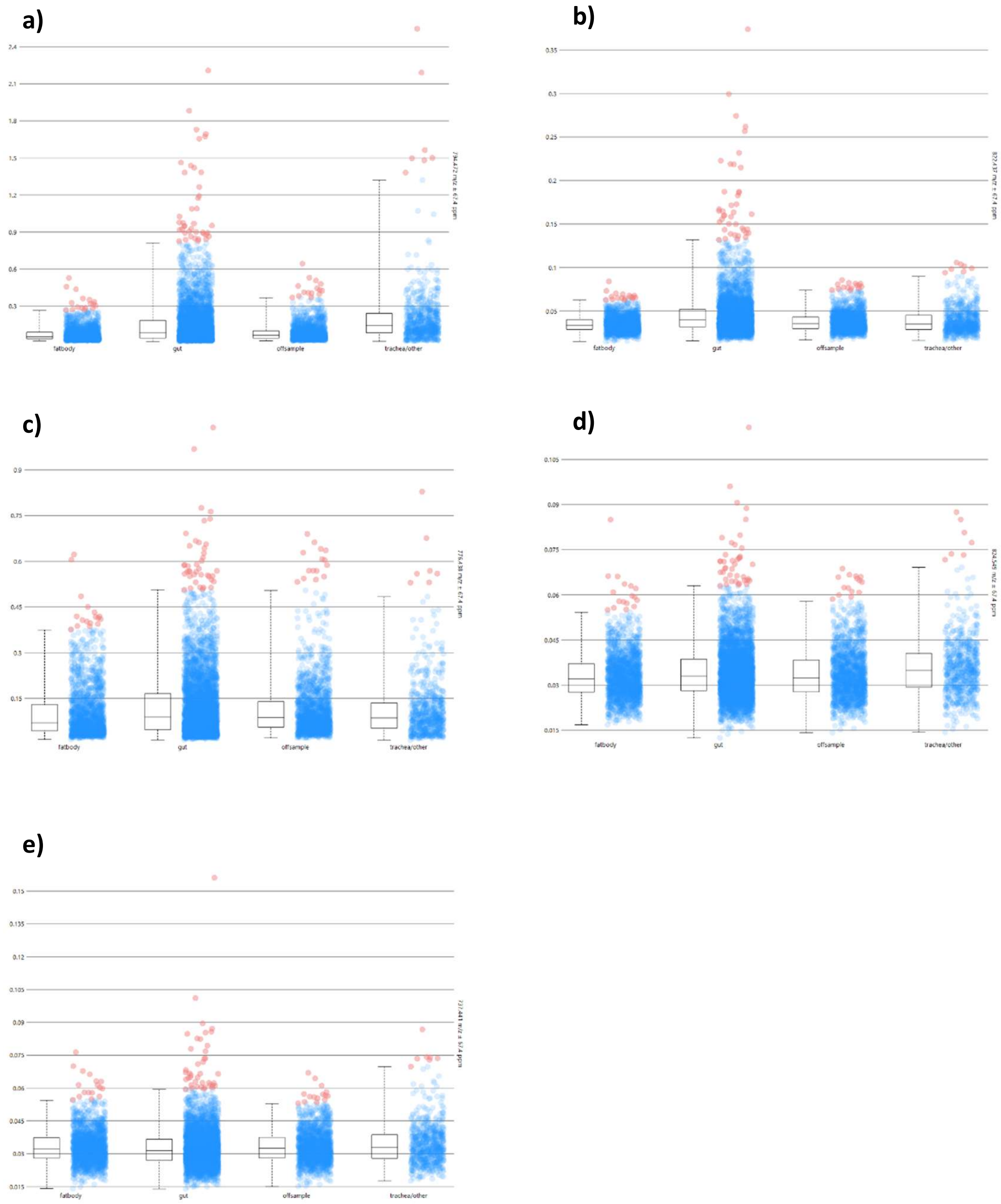

Supporting Figure S15. Intensity box plots for different sample regions shown in Fig. S12 of a) m/z 794.514; b) m/z 822.553; c) m/z 776.498; d) m/z 824.587; e) m/z 737.474 co-localized to gut region.

m/z-values co-localized to trachea/other region

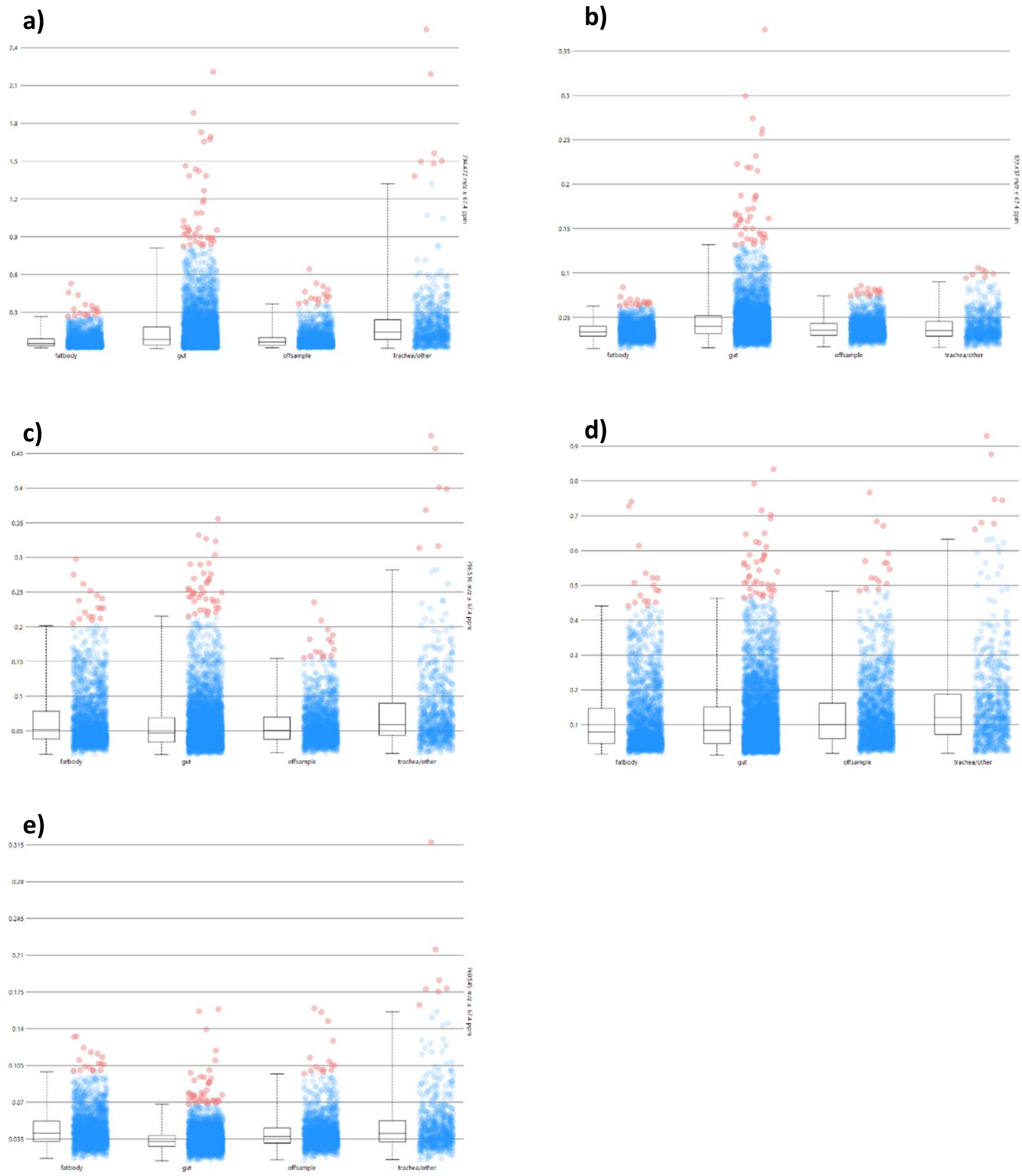

Supporting Figure S16. Intensity box plots for different sample regions shown in Fig. S12 of a) m/z 794.514; b) m/z 822.553; c) m/z 796.552; d) m/z 778.512; e) m/z 760.617 co-localized to trachea/other region.

Segmentation for Dissection No 4 without washing with water

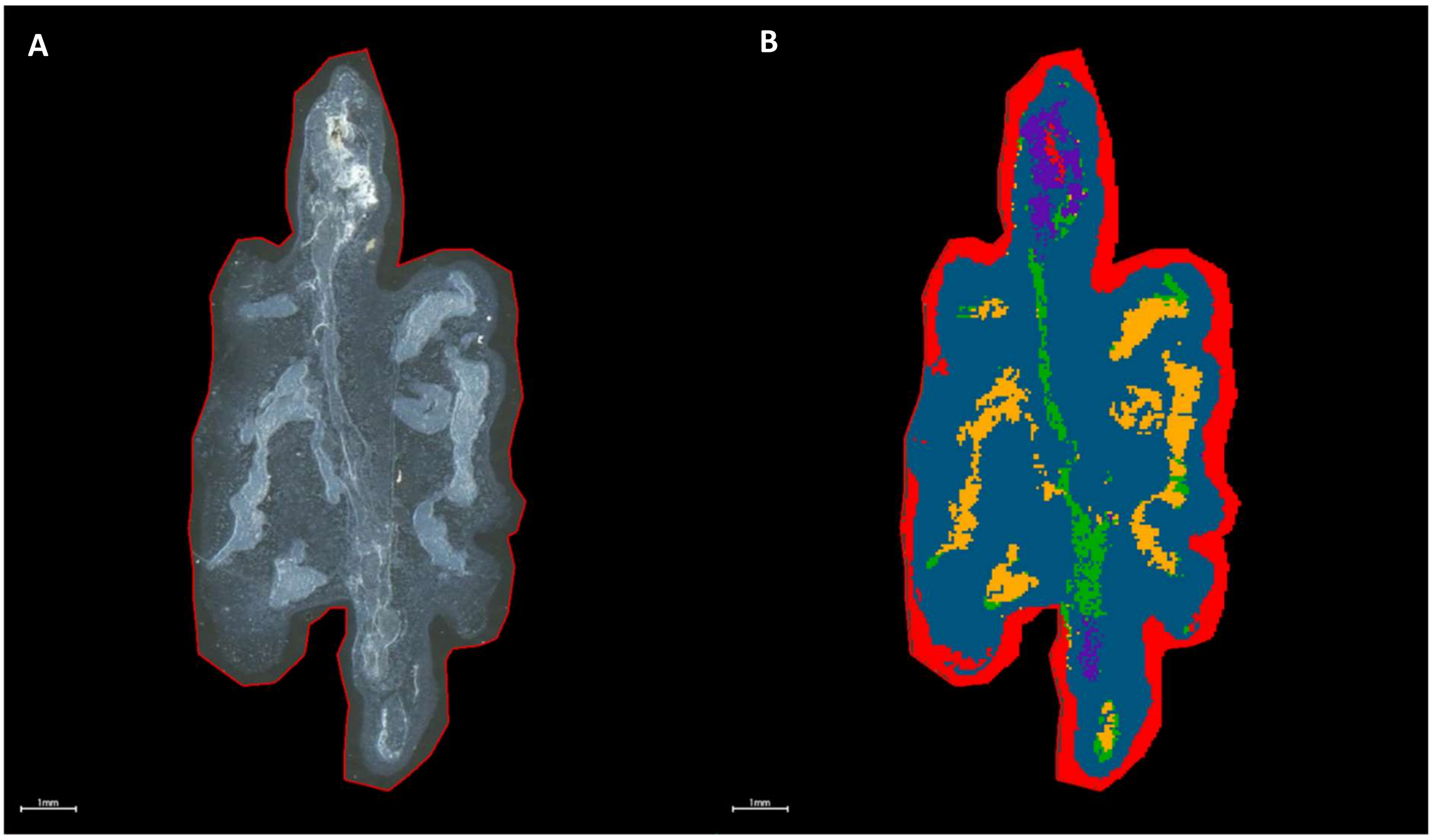

Supporting Figure S17. A) Optical image of dissected Drosophila *larva. B) Clusters from Segmentation Pipeline, measurement after MALDI imaging preparation without the washing step with* ddH_2O
